# Factors underlying surrogate medical decision-making in middle eastern and east Asian women: a Q-methodology study

**DOI:** 10.1186/s12904-020-00643-9

**Published:** 2020-09-01

**Authors:** Muhammad M. Hammami, Areej Al Balkhi, Sophia S. De Padua, Kafa Abuhdeeb

**Affiliations:** 1 Clinical Studies and Empirical Ethics Department, King Faisal Specialist Hospital and Research Centre, P O Box # 3354 MBC 03, Riyadh, 11211 Saudi Arabia; 2 Alfaisal University College of Medicine, Riyadh, Saudi Arabia

**Keywords:** Surrogate medical decision-making, Middle eastern, East Asian, Q-methodology, Averaging-analysis, Norm perception, Patient preference, And surrogate decision-maker preference

## Abstract

**Background:**

It is not clear how lay people prioritize the various, sometimes conflicting, interests when they make surrogate medical decisions, especially in non-Western cultures. The extent such decisions are perspective-related is also not well documented.

**Methods:**

We explored the relative importance of 28 surrogate decision-making factors to 120 Middle-Eastern (ME) and 120 East-Asian (EA) women from three perspectives, norm-perception (N), preference as patient (P), and preference as surrogate decision-maker (S). Each respondent force-ranked (one to nine) 28 opinion-items according to each perspective. Items’ ranks were analyzed by averaging-analysis and Q-methodology.

**Results:**

Respondents’ mean (SD) age was 33.2 (7.9) years; all ME were Muslims, 83% of EA were Christians. “Trying everything possible to save patient,” “Improving patient health,” “Patient pain and suffering,” and/or “What is in the best interests of patient” were the three most-important items, whereas “Effect of caring for patient on all patients in society,” “Effect of caring for patient on patients with same disease,” and/or “Cost to society from caring for patient” were among the three least-important items, in each ME and EA perspectives. P-perspective assigned higher mean ranks to family and surrogate’s needs and burdens-related items, and lower mean rank to “Fear of loss” than S-perspective (*p*<0.001). ME assigned higher mean ranks to “Medical facts” and “Surrogate own wishes for patient” and lower mean rank to “Family needs” in all perspectives (*p*<0.001). Q-methodology identified models that were relatively patient’s preference-, patient’s religious/spiritual beliefs-, or emotion-dependent (all perspectives); medical facts-dependent (N- and S-perspectives), financial needs-dependent (P- and S-perspectives), and family needs-dependent (P-perspective).

**Conclusions:**

1) Patient’s health was more important than patient’s preference to ME and EA women; society interest was least important. 2) Family and surrogate’s needs/ burdens were more important, whereas fear of loss was less important to respondents as patients than as surrogate decision-makers. 3) Family needs were more important to EA than ME respondents, the opposite was true for medical facts and surrogate’s wishes for patient. 4) Q-methodology models that relatively emphasized various surrogate decision-making factors overlapped the ME and EA women’ three perspectives.

## Background

It is not clear how lay people prioritize the various, sometimes conflicting, interests when they make surrogate medical decisions, especially in non-Western cultures. In addition, the extent such decisions are perspective-dependent is not well documented.

According to extended autonomy/precedent consent, a prevalent thesis in Western cultures, surrogate decision-makers are expected to make substituted judgments based on stated or predicted patient’s wishes [1, 2]. However, surrogates may base their decisions, at least in part, on what they think is in the patient’s best interests; what they themselves would have wanted given the circumstances; their needs, burdens, and religious/ spiritual beliefs; and/or their family needs, including maintaining family cohesion. Further, surrogates’ decisions may be emotion-driven such as by fear of loss, feeling of guilt, and desire to pursue any chance of recovery [3–6]. Furthermore, the patient’s own wishes may be difficult to predict because they may be circumstance-dependent and may be influenced not only by consequences to the patient but also to the surrogate, family, friends, and society. A competent patient who wishes not to be intubated may prefer that the surrogate chooses to have him/her intubated if choosing otherwise would place a burden on the surrogate. Moreover, patients may believe that such decisions are not their right, responsibility, or even important to them and thus may not perceive deviations from their preferences as infarctions of their autonomy.

There are several views that disagree with the precedent consent thesis [7–20]. The current preference thesis holds it that one has no moral authority to control their incompetent self and promotes patient’s current interests such as avoiding pain and enjoying simple pleasures. The patient’s life-story narrative models [9–15] focus on respect for persons rather than barely on autonomy and on “authentic” rather than autonomous decisions, aiming to continue the life the patient has led (authentic life model), [14, 15] to promote the life the patient has valued (endorsed life model), [14] or to integrate substituted judgment and best interests standards (substituted interests model) [13, 14]. The family autonomy/familism notion [16–18] argues that the family is the essential social unit and decision-maker, that sickness or end-of-life is a sharing process since they are experienced by the entire family, [18] that the values of patient’s autonomy/beneficence in such situations are related to their importance to someone who loves the patient, [19] and that family’s moral authority is warranted by trust and needs rather than prediction accuracy [18].

End-of-life priorities [21, 22] and factors underlying surrogate decision-making may be culture-dependent; religiosity and social outlook (on liberal-conservative continuum) contributed to the variance in physicians’ attitude toward end-of-life decision-making, [23] and while 85% of the US public expressed desire to be informed if they had < 1 year to live, only 49% of Japanese did [17].

Discrepancies between patients’ perspective and surrogates’ perspective have been noted both in decisions’ content [18, 24–26] and in means of decision-making [27, 28]. Such discrepancies may be related to inadequate knowledge of the patient or to projecting surrogate’s own wishes rather than simulating patient’s wishes [29, 30]. However, they may also be related to the fact that priorities’ hierarchy may be perspective-dependent and that norm-perception may differ from individual preference. Thus, one would consider the relative importance of factors influencing surrogate decision-making from the patient’s perspective, surrogate decision-maker perspective, and norm perception perspective.

Study designs that use independent rating and score-averaging do not provide insight on relative importance [31] and may obscure individual priority structures, respectively [21, 22] In Q-methodology, a forced-ranking study design followed by factor analysis, respondents model their point of view by rank-ordering opinion statements along a fixed continuum of symmetrical distribution [32]. Q-methodology allows assessment of individual priority structures as well as grouping of similar-minded individuals [33].

Using Q-methodology, the primary aim of this study was to explore the relative importance of 28 factors in surrogate decision-making to Middle Eastern and East Asian women from three perspectives, norm-perception, patient’s perspective, and surrogate decision-maker perspective.

## Methods

### Design

This is a cross-sectional study on the relative importance of 28 surrogate decision-making factors to Middle-Eastern (ME) and East-Asian (EA) women from three perspectives: norm perception (N-perspective), preference as patient (P-perspective), and preference as surrogate decision-maker (S-perspective). A Q-methodology instrument was used because it is especially suited to examine relative importance. Briefly, Q-methodology involves construction/collection of a concourse of opinion statements related to the topic under study, sorting the statements into groups and collapsing them into a Q-set that adequately covers the various thematic domains, presenting the Q-set to respondents to model their point of view by rank-ordering the statements into piles (Q-sort) along a continuum defined by certain instructions, performing a special type of by-person exploratory factor analysis using the Q-sorts as variables, grouping of respondents who rank-ordered the statements into similar arrangements (i.e., loaded significantly on the same factor) into models, and interpreting the models [32, 33].

### Sample / setting

Eligibility criteria were having a ME or EA ancestry, age > 18 years, >high school education, and ability to understand study aim and procedures as subjectively determined by study coordinator. Volunteers were recruited through advertisement within Riyadh, Saudi Arabia. The study involved both men and women; however, because of the extent of data, only the results of women are reported here. A sample size of 120 ME and 120 EA women was based on convenience and limitation of the Q-methodology program (maximum of 120 Q-sorts).

### Protection of human subjects / ethical considerations

The study was conducted in accordance with the Declaration of Helsinki after approval of the Research Ethics Committee of the King Faisal Specialist Hospital and Research Center (KFSH&RC) and obtaining respondents’ written informed consent.

### Data collection

Each respondent performed Q-sorting according to each of the three perspectives (3 Q-sorts), in three random same-day Q-sorting sessions. The sessions included writing comments on the 6 extreme selections and were separated by respondents’ completion of 1) respondents’ characteristics sheet and 2) a previously published social value scale questionnaire [23].

Each of the three Q-set consisted of 28 factors that may potentially underline surrogate medical decision-making (items), 16 of which were reported previously [5, 6] and the rest were identified from literature review [3, 13–15]. The 28 items are listed in Additional file 1-Q-set items together with their abbreviated version that is used in the text. The items can be divided into eight domains, patient’s preference-centric (3 items plus one item shared with patient’s life-long narrative-centric), patient’s life-long narrative-centric (4 items plus one item shared with patient’s preference-centric), patient’s health-centric (4 items plus one item shared with surrogate’s emotion-centric), surrogate’s emotion-centric (2 items plus one item shared with patient’s health-centric), surrogate’s preference-centric (3 items), surrogate’s interest-centric (2 items plus one item shared with family-centric), family-centric (4 items plus one item shared with surrogate’s interest-centric), and society-centric (3 items). The wording of the 28 items (as well as the associated instructions) in the three Q-sets were modified to fit the three perspectives addressed in the study. The Q-sets were subjected to two cycles of pilot testing, validation, and revision as previously described [34]. Q-set items were randomly numbered.

Q-sets were given to respondents with sets of instructions and sorting sheets (Additional file 2-instructions and sorting sheets). Q-sorting requires respondents to arrange the items according to their subjective relative importance. In the current study, this was from 1 = least important to 9 = most important. Numbers of slots for each category were symmetrically distributed (Additional file 2-instructions and sorting sheets); for example, there was one slot under categories 1 and 9 and 2 slots under categories 2 and 8. The time spent in Q-sorting was recorded. Sorting sheets were checked for completion (i.e., each item number is used and only once) and were collected before respondents started the next Q-sorting. In case a mistake was found in copying items’ numbers onto the sorting sheet, respondents were notified to self-correct.

Respondents’ characteristics included age, nationality, employment status, self-rated religiosity, major religious affiliation, self-rated health, daily self-care status, pain in last month, pain interference with daily activities, life quality, life satisfaction, living arrangements, death in immediate family/close friends, and personal experience with surrogate decision-making.

The social value scale [23] consisted of three subscales: planning social change (subscale-1, 3 “conservative” and 3 “liberal” statements), free choice regarding medical ethical questions (subscale-2, 4 “conservative” and 3 “liberal” statements), and deciding good vs bad regarding personal welfare (subscale-3, 3 “conservative” and 3 “liberal” statements). Respondents were asked to score each statement from (1 = total disagreement to 5 = full agreement). To analyze responses, we multiplied “liberal” statement scores by − 1 and then calculated the average score for each subscale. Thus, score of subscale-1 could range from − 2 (extreme liberal) to 2 (extreme conservative), of subscale-2 from − 1.57 (extreme liberal) to 2.43 (extreme conservative), and of subscale-3 from − 2 (extreme liberal) to 2 (extreme conservative).

### Data analysis

Q-sorts were analyzed separately for ME respondents and EA respondents and for each of the three perspectives, using PCQ for Windows (PCQ Software, Portland, OR, USA). Data analysis in Q-methodology requires a special program and involves exploring Q-sorts’ correlation, factor analysis (factor loading and rotation), factor scores computation, and factor interpretation. The PCQ program identified and used Q-sorts with a loading of ≥0.49 (*p* < 0.01) on a single factor to create model Q-sorts, which indicate how a respondent with 100% loading on a factor would have ordered the 28 items. Model Q-sorts were interpreted based on the 3 most important (i.e., items assigned to categories 8 and 9) and 3 least important items (i.e., items assigned to categories 1 and 2), respondents post-sorting comments, and relative importance of all items within and across models. Association between models and respondents’ characteristics was studied after grouping respondents who loaded significantly on one model and respondents with confounded loading who had higher loading on the same model. For averaging-analysis, items were considered “neutral” if their mean ranking score was >4 and <6. For Q-methodology, items were considered strongly-agreeable (most important), agreeable, disagreeable, or strongly-disagreeable (least important), if they received a model Q-set rank of 8–9, 7, 3, or 1–2, respectively. Bivariate analysis was performed using chi-square or Fisher Exact tests for categorical variables and ANOVA or t-test for continuous variables (IBM SPSS Statistics version 21 software). An unadjusted two-tailed *p*-value is reported and considered significant if <0.01.

## Results

Table 1 summarizes respondents’ characteristics. All ME respondents were Muslims and 56% were Saudis, whereas 83% of EA respondents were Christians and 94% were Filipinos. ME respondents took less time in Q-sorting with a mean (95% confidence interval) time difference of − 6.2 (− 10.3 to − 2.1) minutes (*p* = 0.003); had more liberal score on social value subscale-1 with a mean difference of − 0.20 (− 0.32 to − 0.08) on a scale from − 2.0 to 2.0 (*p* = 0.001) and more conservative score on social value subscale-2 with a mean difference of 0.32 (0.19 to 0.46) on a scale of − 1.57 to 2.43 (*p*<0.001); and reported better self-rated health (*p* = 0.01), higher life quality (p = 0.001), lower life satisfaction (*p*<0.001), lower employment rate (*p*<0.001), more recent experience with surrogate decision-making (*p* = 0.009), and different living arrangement (*p*<0.001). Differences in other characteristics were not statistically significant. Responses to individual social value scale statements are presented in Table 1S (Additional file 3-social value scale).

**Table 1:** Characteristics of study respondents

	Middle Eastern (***n*** = 120)	East Asian (***n*** = 120)
**Age-mean (SD), yr.**	32.7 (9.4)	33.7 (6.0)
**Nationality- no. (%)**		
Saudi	67 (56)	–
Filipino	–	113 (94)
Syrian	23 (19)	–
Others	30 (25)	7 (6)
**Employment status- no. (%)**		
Student	23 (19)	0 (0)
Employed	83 (69)	119 (99)
Self employed	3 (3)	1 (1)
Not employed	3 (3)	0 (0)
**Self-rated religiosity- no. (%)**		
1 (least)	2 (2)	3 (3)
2	6 (5)	1 (1)
3	65 (54)	52 (43)
4	34 (28)	47 (39)
5 (most)	13 (11)	17 (14)
**Religious affiliation no. (%)**		
Islam	120 (100)	19 (16)
Christianity	0 (0)	99 (83)
Others	0 (0)	2 (2)
**Social value subscales-mean (SD)**		
1.Planning social change*	−0.33 (0.52)	−0.14 (0.40)
2.Free choice regarding medical ethical questions**	0.64 (0.57)	0.31 (0.48)
3.Deciding good vs bad regarding personal welfare***	0.01(0.48)	0.04 (0.50)
**Self-rated health- no. (%)**		
Excellent	36 (30)	18 (15)
Very good	52 (43)	70 (58)
Good	27 (23)	31 (26)
Fair	4 (3)	1 (1)
Poor	1 (1)	0 (0)
**Daily self-care-no. (%)**		
Without help	118 (98)	119 (99)
With some help	2 (2)	1 (1)
Completely unable to do any housework	0 (0)	0 (0)
**Pain in last month-no. (%)**		
None	34 (28)	48 (40)
A little bit	57 (48)	46 (38)
Moderate	22 (18)	19 (16)
Quite a bit	4 (3)	7 (6)
Extreme	3 (3)	0 (0)
**Pain interference with daily activities-no. (%)**		
Not at all	71 (59)	79 (66)
A little bit	34 (28)	24 (20)
Moderately	10 (8)	13 (11)
Quite a bit	4 (3)	4 (3)
Extremely	1 (1)	0 (0)
**Current Quality of life-no. (%)**		
Excellent	39 (33)	18 (15)
Very good	56 (47)	81 (68)
Good	25 (21)	19 (16)
Fair	0 (0)	2 (2)
**“If I could live my life over, I would change almost nothing.”-no. (%)**		
Strongly agree	12 (10)	22 (18)
Agree	25 (21)	56 (47)
Neither agree nor disagree	23 (19)	31 (26)
Disagree	33 (28)	10 (8)
Strongly disagree	27 (23)	1 (1)
**Living arrangements-no. (%)**		
With spouse	46 (38)	54 (45)
With parent	60 (50)	13 (11)
With child	6 (5)	2 (2)
With other family members	7 (6)	8 (7)
Alone	1 (1)	43 (36)
**Death in immediate family/close friends-no. (%)**		
Last year	24 (20)	26 (22)
2–5 years ago	55 (46)	46 (38)
None in last 5 years	41 (34)	48 (40)
**Personal experience with surrogate decision-making-no. (%)**		
Last year	34 (28)	14 (12)
2–5 years ago	21 (18)	21 (18)
Six or more years ago	4 (3)	7 (6)
None	61 (51)	78 (65)
**Sorting time-mean (SD), min.**	67.2 (14.5)	73.4 (17.6)

Percentages refer to number of responses and may not add to 100% due to rounding. *Subscale-1 ranges from −2.0 (most liberal) to 2.0 (most conservative). **Subscale-2 ranges from −1.57 (most liberal) to 2.43 (most conservative). ***Subcale-3 ranges from − 2.0 (most liberal) to 2.0 (most conservative). Responses to individual social value scale statements are presented in Table 1S under Additional file 3-social value scale

### Averaging-analysis

**Fig. 1:**
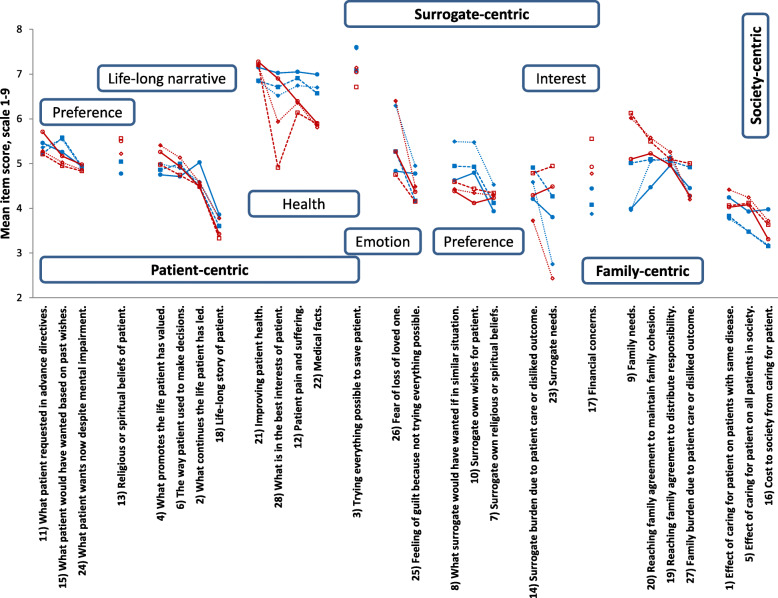
Forced-ranking scores of 28 opinion-items related to surrogate medical decision-making. The items are arranged according to the most relevant underlying domain. Data represent mean ranking scores on a scale of 1 (least important) to 9 (most important). Closed blue and open red symbols refer to Middle-Eastern and East-Asian women, respectively. Circles and solid lines indicate norm-perception, squares and interrupted lines indicate patient’s perspective, and diamonds and dotted lines indicate surrogate decision-maker perspective. For full description of the items, see Additional file 1-Q-Set items

Figure 1 shows mean ranking scores of the 28 items by ME and EA respondents from the N-perspective, P-perspective, and S-perspective. The items are grouped according to the underlying domains.

Overall, patient’s health was more important than other factors, including patient’s preference and life-long narrative; surrogate’s emotion, preference, and interest; family interests; and society interests. For ME respondents, of the items that received a mean ranking score >6, 5/5, 5/5, and 5/6 were related to patient’s health in the N-perspective, P-perspective, and S-perspective, respectively. For EA respondents, the corresponding ratios were 4/4, 3/4, and 3/5. The other items that received a mean score >6 were “26.Fear of loss of loved one” in ME and EA S-perspective and “9.Family needs” in EA P-perspective and S-perspective.

Notably: 1) In the patient’s preference domain, patients’ wishes were given about the same weights regardless of patients’ mental status. 2) In the patient’s life-long narrative domain, the weight given to “18.Life-long story of patient” was lower than the weight given to the other four related items, suggesting that it may have not been understood correctly by respondents. 3) In the patient’s health domain, “3.Trying everything possible to save patient” received more weight than “22.Medical facts.” 4) In the surrogate’s preference domain, there were no appreciable differences between the weights given to what surrogates want for themselves or wish for patients.

#### Comparing the three perspectives

There were significant (*p*<0.001 to *p* = 0.006) differences among the N-, P-, and S-perspectives in mean scores of several items, when ME and EA respondents were analyzed together, ME respondents analyzed alone, or EA respondents analyzed alone (Table 2). Of note: 1) Surrogate’s and family’s needs and burdens were given more weight in the P-perspective than the S-perspective. 2) Fear of loss, feeling of guilt, and patient’s interests were given more weight in the S-perspective than the P-perspective. 3) There was dissociation between the N-perspective and S-perspective in relation to importance of surrogate’s preference and family cohesion (S>N), and patient’s best interests (N>S).

**Table 2:** Surrogate decision-making items with significant differences in ranking scores among three perspectives

Item	All	Only ME	Only EA
“28.What is in the best interests of patient”	N>S>P		N>S>P
“26.Fear of loss of loved one”	S>N & P	S>P & N	S>N & P
“25.Feeling of guilt because not trying everything possible”	S & N>P	S & N>P	
“8.What surrogate would have wanted if in similar situation”	S>N	S>N & P	
“10.Surrogate own wishes for patient”	S>N	S>P & N	
“14.Surrogate burden due to taking care of patient or disliked outcome”	P>N & S	P>N	P>N>S
“23.Surrogate needs”	P>N>S	P & N>S	P & N>S
“9.Family needs”	P>S>N	P>S & N	P & S>N
“20.Reaching family agreement to maintain family cohesion”	S & P>N	P & S>N	
“27.Family burden due to taking care of patient or disliked outcome”	P>N & S	P>S	P>N & S

Items’ ranks were compared among the N, P, and S perspectives (norm perception, patient, and surrogate decision maker perspectives, respectively) in all respondents (All), only Middle Eastern (ME) respondents, and only East Asian (EA) respondents. Items with significant (*p* < 0.01) rank differences are shown, grouped according to the underlying domain. *P* values ranged from 0.006 to < 0.001

#### Comparing ME to EA women

Similarly, there were significant (p<0.001 to *p* = 0.008) differences between ME and EA respondents in mean ranking scores of several items (Table 3). In all three perspective, ME respondents were more influenced by patient’s health and less influenced by family needs. Further, ME respondents gave more weight to patient’s past wishes in the P-perspective. Furthermore, there was differential emphasis on the various aspects of the patient’s life-long narrative; ME respondents gave higher score to the life the patient has led (N-perspective) and lower score to the life the patient has valued (N-perspective and S-perspective) and religious or spiritual beliefs of patient (N-perspective). Finally, EA respondents gave more weight to society interests in the P-perspective and S-perspective.

**Table 3:** Surrogate decision-making items with significant differences in ranking scores between ME and EA women

Items	N-perspective		P-perspective		S-perspective	
	ME	EA	ME	EA	ME	EA
“15.What patient would have wanted based on past wishes”			x		x	
“13.Religious or spiritual beliefs of patient”		x				
“4.What promotes the life patient has valued”		x				x
“2.What continues the life patient has led”	x					
“28.What is in the best interests of patient”			x		x	
“12.Patient pain and suffering”	x		x			
“22.Medical facts”	x		x		x	
“3.Trying everything possible to save patient”	x					
“8.What surrogate would have wanted if in similar situation”					x	
“10.Surrogate own wishes for patient”	x		x		x	
“14.Surrogate burden due to taking care of patient or disliked outcome”					x	
“23.Surrogate needs”		x		x		
“17.Financial concerns”				x		x
“9.Family needs”		x		x		x
“20.Reaching family agreement to maintain family cohesion”		x				x
“1.Effect of caring for patient on patients with same disease”						x
“5.Effect of caring for patient on all patients in society”				x		x

Items’ ranks were compared between Middle Eastern (ME) and East Asian (EA) women per perspective. N-, P-, and S- perspectives indicate norm perception, patient, and surrogate decision-maker perspectives, respectively. Items with significant (*p* < 0.01) rank differences are shown, grouped according to the underlying domain. *P* values ranged from 0.008 to < 0.001. “x” indicates the group with higher mean rank

### Q-methodology analysis

Using a Q-methodology specific program (PCQ program), we extracted 6 factors for each of the three perspectives of ME and EA respondents. The program calculates idealized item scores (model Q-sort) for each factor. A model Q-sort indicates how a respondent with 100% loading on a factor would have ordered the 28 items. Factor characteristics and item scores are presented in Tables 2S to 7S (Additional file 4-factor characteristics and item scores).

The final step in Q-methodology is to interpret and name program-identified model Q-sorts and explore their association with respondents’ characteristics. Tables 4 and 5, list model names in each perspective of ME and EA respondents, respectively. Details underlying factors interpretation are presented in Additional file 5-Q-methodology technical report. The following provides an overall description of models in each perspective.

**Table 4:** Surrogate medical decision-making models in Middle Eastern women

Norm-perception (N-perspective) models	
Model A: Surrogate’s burden-independent	
Model B: Surrogate-independent	
Model C: Emotion, patient’s life-long narrative, and family needs-independent	
Model D: Patient’s endorsed-life narrative and Golden Rule-independent	
Model E: Patient’s preference-dependent	
Model F: Society’s interests-independent	
**Patient’s perspective (P-perspective) models**	
Model A: Emotion and patient’s life-long narrative-independent	
Model B: Cost and society’s interests-independent	
Model C: Emotion-dependent and society’s interests-independent	
Model D: Religious/spiritual beliefs-independent and family needs-dependent	
Model E: Emotion-independent and family needs-dependent	
Model F: Patient’s preference-dependent	
**Surrogate’s perspective (S-perspective) models**	
Model A: Patient’s preference-dependent	
Model B: Emotion and religious/spiritual beliefs-dependent	
Model C: Society’s interests and religious/spiritual beliefs-independent	
Model D: Patient’s life-long narrative-independent	
Model E: Medical facts-dependent and religious/spiritual beliefs-independent	
Model F: Society’s interests-independent	

Models interpretation details are presented in Additional file 5 Q-methodology technical report

**Table 5:** Surrogate medical decision-making models in East Asian women

Norm-perception (N-perspective) models	
Model A: Society’s interest and surrogate-independent	
Model B: Religious/spiritual beliefs and surrogate-independent and emotion-dependent	
Model C: Medical facts-dependent and patient’s preference independent	
Model D: Financial needs and society’s interests-independent	
Model E: Patient’s preference-dependent and emotion-independent	
Model F: Patient’s religious/spiritual beliefs-dependent	
**Patient’s perspective (P-perspective) models**	
Model A: Society’s interests-independent	
Model B: Religious/spiritual beliefs-independent	
Model C: Financial needs-dependent and emotion-independent	
Model D: Emotion-independent and family needs-dependent	
Model E: Patient’s preference-independent and patient’s religious/spiritual beliefs-dependent	
Model F: Patient’s preference-independent	
**Surrogate’s perspective (S-perspective) models**	
Model A: Religious/spiritual beliefs and financial needs-independent	
Model B: Society’s interest-independent	
Model C: Family burden-independent and religious/spiritual beliefs-dependent.	
Model D: Surrogate’s preference-independent	
Model E: Financial needs-dependent and patient’s authentic-life narrative-independent	
Model F: Society’s interests-independent and religious/spiritual beliefs-dependent	

Models interpretation details are presented in Additional file 5 Q-methodology technical report

#### Norm-perception models of ME women

There were three consensus items among the six models in this perspective, and all were related to patient’s health: “3.Trying everything possible to save patient,” “21.Improving patient health,” and “28.What is in the best interests of patient” (ranked 8 or 9, 7 or 8, and 7 or 8, respectively). However, ranks for “22.Medical facts” ranged from 6 to 9. Further, ranks of the six items with a mean score <4 on averaging-analysis varied from 1 to 6 for “7.Surrogate own religious or spiritual beliefs,” from 1 to 5 for “23.Surrogate needs,” from 2 to 6 for “9.Family needs,” from 1 to 5 for “18.Life-long story of patient,” from 1 to 7 for “16.Cost to society from caring for patient,” and from 3 to 5 for “5.Effect of caring for patient on all patients in society.” The results indicate more heterogeneity in what is considered unimportant compared to what is considered important.

Despite the overall emphasis on patient’s health, there was a relatively patient’s preference-dependent model (Table 4). We found no significant associations between respondent characteristics and loading on a given model in this perspective.

#### Patient’s perspective models of ME women

In this perspective, none of the patient’s health items received a consensus status. In fact, ranks for “28.What is in the best interests of patient” and “12.Patient pain and suffering” varied from 6 to 9. On the other hand, “1.Effects of caring for patient on patients with same disease” was a consensus item (ranked 3 or 4).

This perspective was notable for having two relatively family needs-dependent models, a relatively patient’s preference-dependent model, and a relatively emotion-dependent model (Table 4). Further, model loading was associated with age (*p* = 0.001), employment status (*p* = 0.006), and living arrangements (*p*<0.001). Models A and B respondents were older than models D and C respondents (mean (SD) age 38.9 (10.0), 36.4 (11.4), 25.9 (6.9), and 25.8 (3.5) years, respectively). Further, model C (unique in this perspective in being relatively emotion-dependent) was composed mainly of students (56% vs 0 to 22% in other models). In addition,

86% of model A (unique in being relatively patient’s life-long narrative-independent) respondents lived with a spouse (vs 0–33% in other models) and 89% of model D (unique in being relatively religious/spiritual beliefs-independent) respondents lived with a parent (vs 14–75% in other models).

#### Surrogate decision-maker perspective models of ME women

In this perspective, “1.Effects of caring for patient on patients with same disease” was a consensus item with low ranks (ranked 3 or 4), “8.What surrogate would have wanted if in similar situation” was a consensus item with intermediate ranks (ranked 5 or 6), and “21.Improving patient health” was a consensus item with high/very high ranks (ranked 7 or 8).

On the other hand, ranks of several items varied considerably; from 5 to 8 for “22.Medical facts,” from 4 to 9 for “26.Fear of loss of loved one,” from 2 to 7 for “17.Financial concerns” and “9.Family needs,” and from 1 to 5 for “18.Life-long story of patient.”

This perspective included a relatively emotion and religious/spiritual beliefs-dependent model, a relatively medical-facts-dependent model, and a relatively patient’s preference-dependent model (Table 4). Association with model loading was restricted to social value subscales-1 and subscale-2 scores and was of borderline significance (*p* = 0.02). Model A (unique in being relatively patient’s preference-dependent) had “more liberal” mean score than the relatively society’s interests-independent model F on subscale-1 (− 0.70 (0.69) vs − 0.14 (0.59) and “less conservative” mean score on subscale-1 (0.27 (0.71) vs 0.91 (0.61) on subscale-2.

#### Norm-perception models of EA women

“21.Improving patient health” was a consensus item (ranked 8 or 9) in this perspective. However, ranks of the other patient’s health related items varied from 5 to 9 for “28.What is in the best interests of patient” and from 6 to 9 for “3.Trying everything possible to save patient” and “12.Patient pain and suffering.” This perspective included relatively emotion-dependent, medical facts-dependent, patient’s preference-dependent, and patient’s religious/spiritual beliefs-dependent models (Table 5). Similar to the case in ME women N-perspective, there was no significant association between respondent characteristics and model loading.

#### Patient’s perspective models of EA women

In this perspective, “9.Family needs” was one of the four items with a mean score of >6 on averaging-analysis and was ranked 6 to 8. The other 3 items were again related to patient’s health. “21.Improving patient’s health” was a consensus item (ranked 8 or 9); however, ranks varied from 3 to 8 for “12.Patient’s pain and suffering” and from 5 to 9 for “3.Trying everything possible to save the patient.”

Notably, this perspective included relatively financial needs-dependent, family needs-dependent, and patient’s religious/spiritual beliefs-dependent models (Table 5). Further, there was significant (*p* = 0.007) association between model loading and social value subscale-3 scores. Model D (unique in being family needs-dependent) had “more conservative” mean score than models B and E (0.48 (0.61) vs − 0.14 (0.19) and − 0.11 (0.38), respectively). Model B was relatively religious/spiritual beliefs-independent, whereas model E was relatively patient’s religious/spiritual beliefs-dependent.

#### Surrogate decision-maker perspective models of EA women

In this perspective, “21.Improving patient health” was a consensus item (ranked 8 or 9). However, ranks of several items varied widely, including from 5 to 9 for “26.Fear of loss of loved one,” from 6 to 9 for “3.Trying everything possible to save patient” and “12.Patient pain and suffering,” from 1 to 6 for “16.Cost to society from caring for patient,” from 2 to 5 for “14.Surrogate burden due to taking care of patient or disliked outcome.”

There were two relatively religious/spiritual beliefs-dependent models and a relatively financial needs-dependent model in this perspective (Table 5). There was borderline significant (*p* = 0.04) association between factor loading and social value subscale-3 scores. Model E (unique in being relatively patient’s authentic-life narrative-independent) had more “liberal” score on subscale-3 than the relatively society’s interest-independent model B (− 0.13 (0.37) vs 0.30 (0.55), respectively).

## Discussion

The primary aim of this study was to explore the relative importance of 28 factors in surrogate medical decision-making by ME and EA women. The factors covered the following areas: patient’s preference, life-long narrative, and health; surrogate’s emotion, preference, and interests; family interests; and society interests. Three perspectives were examined, norm-perception, patient’s perspective, and surrogate decision-maker perspective. Our main findings were: 1) Patient’s health was more important than patient’s preference to both ME and EA women in all three perspectives. 2) Respondents put more weight on family and surrogate’s needs/ burdens and less weight on fear of loss as patients than as surrogate decision-makers. 3) Family needs were more important to EA than ME women; the opposite was true for medical facts and surrogate’s wishes for patient. 4) ME and EA women could be assigned to heterogeneous but overlapping Q-methodology models that were associated with a few of respondents’ characteristics.

### Importance of patient’s health

According to the substituted judgment standard, surrogate decisions should be based on patient’s stated or predicted preferences rather than what the surrogate thinks is in the patient’s best interests, which is supported by the results of a national survey of US physicians [2]. However, in the current study, patient’s health was more important than other studied factors, including patient’s preference, for both ME and EA respondents and from all the three perspectives. Consistently, a previous study using simple rating and dichotomization showed that 78% of surrogates (mostly white educated US women) focused more on patient’s well-being than patient’s preferences [6]. A predominance of consequentialist attitude over autonomy-based attitude was also observed in lay people approach to medical use of placebos [34] and organ donation, [35] providing support to the concept that “good” may be more fundamental than “right” and to the importance of “harm/care” as one of the psychological foundations of morality [36].

On the other hand, placing more weight on patient’s health than patient’s preference may be emotion-driven. Indeed, for our respondents, trying everything possible to save patient was more important than other patient’s health-related items such as medical facts and patient’s best interests (Figure). Substituted judgement is known to be associated with emotional demands, [18] and about a third of surrogates had negative emotions lasting several months [37]. Alternatively, the emphasis on patient’s health over patient’s preference may be related to beliefs of life sacredness.

### Patient’s preference and life-long narrative

Overall, patient’s preference followed patient’s health in importance. Remarkably, current wishes (despite impaired mentation) were given similar weights to past wishes in most perspectives (Figure). The concept of prospective autonomy/ autonomy extension argues that desiring is only a basic notion, whereas valuing is a reflective appraisal that should be respected when previous and current desires conflict, [8] that autonomy is a right rather than necessarily a reflective capacity, that legitimate interests include not only experiential but also critical interests (such as interests in fulfilling religious commitments and interests in family welfare after death), and that personal identity/personhood is based on bodily continuity rather than psychological connectedness or continuity [7, 8]. In contrast, the current preference thesis takes into account only experiential interests, believes that preferences and interests do not survive loss of mental capacity, considers autonomy as reflective capacity/responsibility, and concludes that there is no moral authority for exercising control over one’s incompetent self and that one should instead promote the current interests of patients [7, 8]. The current preference thesis could provide yet another explanation for our respondents’ emphasis on patient’s health rather than patient’s preference.

Several of the items related to patient’s life-long narrative received similar weights as items related to patient’s preference. According to the narrative view, narratively well-structured lives have an aesthetic value, and life is to be concluded in a way that is best consistent with life-long themes [11, 14]. This view focuses on respect for persons rather than narrowly on respect for autonomy and on authentic decisions rather than autonomous decisions. In addition to taking into consideration patient’s prior wishes, it considers patient’s life-long dispositions, relationships, decisions, decision-making processes, and interests, balancing rather than prioritizing [9]. There are three closely-related versions of the life-story narrative model, authentic life, [10, 15] substituted interests, [13] and patient’s endorsed life [14]. The patient’s life-story is taken as evidence of what the patient would have wanted to continue, [15] of what the patient’s interests should be based upon, [13] or of what the patient have valued, respectively [14].

In most perspectives, our respondents put less weight on what continues the life the patient has led (authentic life) than on what promotes the life the patient has valued (endorsed life) or the way the patient used to make decisions (Figure). However, Q-methodology did identify both a relatively patient’s endorsed-life narrative-independent model (ME N-perspective) and a relatively patient’s authentic-life narrative-independent model (EA S-perspective). Finally, the fact that, in general, the importance of patient’s preference followed the importance of patient’s health appears to be most consistent with the substituted interests’ model, which integrates the substituted judgment and the best interests’ standards [13, 14]. Of note, “Life-long story of patient” was assigned much lower weight than the other items under the life-long narrative domain, suggesting that this item may have not been understood by our respondents as intended.

### The Golden rule

Consistent with the Golden Rule, there were no overall appreciable differences between the importance of “What surrogate would have wanted if in similar situation” and the importance of “Surrogate own wishes for patient.” It has been argued that the Golden Rule, a fundamental concept of Judo-Christian and Islamic ethics, should be considered as a more practical alternative to substituted judgment [5]. Interestingly, the Golden Rule was more important to ME than EA respondents, ME respondents put significantly more weight on surrogate’s wishes for the patient in the N-, P-, and S-perspectives and more weight on what the surrogate would have done in a similar situation in the S-perspective. Finally, the observation that in the ME S-perspective, these two items were as important as items related to patient’s preference provides support to previous results showing that surrogates may more project their wishes than simulate patient’s wishes [29].

### Differences among the three perspectives

In the current study, surrogate/family needs and burdens were given more weight in the P-perspective than in the S-perspective. Similarly, in a previous study, burden on family was the third most important end-of-life issue for patients but was not important for surrogates [38]. Several studies [26, 27, 29, 30, 38] and reviews [24, 25] have found that substituted judgment accuracy is low to moderate despite the fact that surrogates are more accurate than physicians in predicting patients’ preferences [25, 39]. Although this may be caused by inadequate knowledge of patient’s wishes and priorities, [29, 30] the current results suggest that it may be also role-dependent. This is supported by the fact that patient’ best interests and emotions (fear of loss of loved and feeling of guilt) were given more weight in the S-perspective than in the P-perspective.

There was also notable dissociation between the N-perspective and S-perspective. In the N-perspective, respondents put less weight on surrogate’s preference and family cohesion and more weight on patient’s best interest. Previous studies showed significant divergence between preference and perception of norm in regard to consenting for research on medical records and leftover tissue samples [40] but not in regard to disclosure of medical errors [41] or organ donation [42]. One can differentiate two kinds of norm perception. Perception of what is commonly approved/ disapproved (injunctive norm) and perception of what is commonly done (descriptive norm). Our instrument was designed to explore the perception of injunctive norm. The observed dissociation between the N- and S-perspectives suggests that social desirability bias did not have a substantial influence on our respondents.

### Differences between ME to EA respondents

End-of-life preferences may be culture and sex [21, 22, 43] as well as religiosity [23] dependent. We observed two notable differences between ME and EA respondents. First, EA respondents were more family-centric, whereas ME respondents were more patient-centric. In a previous study on attitude to organ donation, there was also stronger familism orientation in Christians/Philippines-educated than Muslims/Saudi Arabia-educated respondents. In familism, which is prevalent in East Asia, [16, 17, 44] the family makes decisions collectively based on the family’s interest [5]. Interestingly, familism orientation may be role-dependent. In our study, family needs and burden were given more weight in the P-perspective compared to the S-perspective. Similarly, a previous study showed that surrogates err more by attributing to the patient a preference for substituted judgment when the patient prefers patient-family shared decision-making or family-only decision-making than the reverse [27].

Second, in the N-perspective, ME respondents put significantly more weight on “What continues the life patient has led” and less weight on “What promotes the life patient has valued” and “Religious or spiritual believes of patient,” suggesting a differential subscription to the authentic- life narrative and endorsed-life narrative models.

### Q-methodology-based surrogate’s decision-making models

Q-methodology, a special type of by-person exploratory factor analysis, produces grouping of respondents into thinking models [32, 33]. Comparing ME and EA models, we found substantial overlap but also notable differences. For example, in the N-perspective, relatively surrogate-independent, society’s interests-independent, and emotion-independent models were found in both groups; patient’s life-long narrative-independent, patient’s endorsed-life narrative-independent, family needs-independent, and Golden rule-independent models were found only among ME respondents; and religious/spiritual beliefs-independent and patient’s preference-independent models were found only among EA respondents. Similarly, in the N-perspective, there were relatively patient’s preference-dependent models in both groups; and emotion-dependent, medical facts-dependent, and patient’s religious/spiritual beliefs-depended models only among EA respondents.

Intriguingly, there was no association between Q-methodology models and characteristics of ME or EA respondents in the N-perspective. However, in the ME P-perspective, respondents who loaded on a relatively emotion-dependent model were younger and predominantly students; in the EA P-perspective, the relatively family needs-dependent model had the most “conservative” score while the relatively religious/spiritual beliefs-independent model had the most “liberal” score on social value subscale-3 (deciding good vs bad regarding personal welfare); in the ME S-perspective, the relatively patient’s preference-dependent model had the most “liberal” score and the relatively society’s interests-independent model had the most “conservative” score on social value subscale-1 (planning social change) and subscale-2 (free choice regarding medical ethical questions); and in the EA S-perspective, the relatively society’s interests-independent model had the most “conservative” scores on social value subscale-3.

### Study potential implications

The results suggest that guidelines for surrogate decision-making that are based solely on right of self-determination may, in general, poorly fit surrogate decision-making in Middle Eastern and East Asian cultures. However, there is no one-size-fits-all solution as patient’s preference-dependent models were identified in both groups. Further, the discrepancy between how one prefers to make surrogate decisions and how one prefers surrogate decisions be made on their behalf should be considered in clinical practice and in designing and interpreting related research studies. Finally, the results highlight the relative importance of familism orientation in surrogate decision-making, especially among East Asian women. It is suggested that clinicians develop measures for eliciting both patients’ and surrogates’ perspectives to include them into the care and education of patients at risk for requiring surrogate decision-making and their potential surrogates.

### Study strengths and limitations

The study was unique in examining an expanded collection of potential factors that may underlie surrogate medical decision-making, in enrolling Middle Eastern and East Asian women, and in analyzing data by both averaging-analysis and Q-methodology. The use of shuffled items and forced-ranking permitted minimizing biases related to order effect and tendency to assign maximum importance to numerous items, respectively. The study design also allowed direct comparison of three perspectives, norm-perception, preference as patient, and preference as surrogate decision-maker. Finally, the three Q-sorts were performed in a random order and separated by completion of respondents’ characteristics and social value scale questionnaire to minimize potential carry-over effect.

The study has several limitations. First, generalizability of the results is limited due to convenience sampling, limiting enrollment to educated individuals, and limiting analysis to women respondents. Second, it is possible that some respondents may have not understood some items as intended (for example, “Life-long story of patient”). Third, it is likely that there are more than the models identified in the study, which is expected because only six factors were extracted and is consistent with the exploratory and none exhaustive nature of Q-methodology. Fourth, given the impressionistic nature of Q-methodology, there was overlap among the models in each perspective.

## Conclusions

We found that in surrogate medical decision-making: 1) Patient’s health was more important than patient’s preference to both Middle Eastern and East Asian women from all three perspectives, norm-perception, preference as patient, and preference as surrogate decision-maker. This is more in line with the substituted interests than the substituted judgment concept. Society interest was least important. 2) Respondents put more weight on family and surrogate’s needs and burdens and less weight on fear of loss as patients than as surrogate decision-makers. 3) Family needs were more important to East Asian than Middle Eastern women; the opposite was true for medical facts and surrogate’s wishes for patient. 4) Middle Eastern and East Asian women could be assigned to heterogeneous but overlapping Q-methodology models that were associated with some respondents’ characteristics and included, relatively patient’s preference-, religious/spiritual beliefs-, emotion-, family needs-, and financial needs- dependent or independent models; patient’s life-long narrative-, surrogate-, Golden Rule-, and society’s interests-independent models; and medical facts-dependent model.

## Supplementary information

**Additional file 1.** Q-set items.

**Additional file 2.** Instructions and sorting Sheet.

**Additional file 3.** Social value scale.

**Additional file 4.** factor characteristics and item scores.

**Additional file 5.** Q-methodology technical report.

## Data Availability

Additional data are available under Supplementary Martial-Additional Files 1 to 5. Raw data are available from the corresponding author upon request.

## Abbreviations

ME: Middle Eastern
EA: East Asian
N-perspective or N: Norm-perception perspective
P-perspective or P: Preference-as-patient perspective
S-perspective or S: Preference-as-surrogate-decision-maker perspective
KFSH&RC: King Faisal Specialist Hospital & Research Center
ANOVA: Analysis of variance
