# Supplementary material for: Factors underlying surrogate medical decision-making in middle eastern and east Asian women: a Q-methodology study
Source: BMC Palliat Care. 2020 Sep 1;19:137. doi: 10.1186/s12904-020-00643-9 (PMC7466416; doi:10.1186/s12904-020-00643-9)
Supplement: Supplementary file 1 — Additional file 1. Q-set items. [file 12904_2020_643_MOESM1_ESM.doc]

**Additional file 1-Q-set items**

**Items according to domains and subdomains**

A, is the abbreviated version used in the text. N, P, and S are full length versions used to study norm, patient, and surrogate perspectives, respectively. The items were assigned random number.

**Patient-centric, preference**

A11) What patient requested in advance directives.

N11) What the patient requested in his/her advance directive.

P11) What you requested in your advance directive.

S11) What my family member requested in his/her advance directive.

A15) What patient would have wanted based on past wishes.

N15) What the patient would have wanted his family to do, based upon his/her wishes in the past.

P15) What you would have wanted your family member to do, based upon your wishes in the past.

S15) What my family member would have wanted our family to do, based upon his/her wishes in the past.

A24) What patient wants now despite mental impairment.

N24) What the patient at-the-time say he/she wants his/her family member to do (despite his /her mental impairment).

P24) What you at-the-time say you want your family member to do (despite your mental impairment).

S24) What my family member at- the-time says he/she wants our family to do (despite his /her mental impairment).

A13) Religious or spiritual beliefs of patient. **^1^**

N13) The religious or spiritual beliefs of the patient.

P13) Your religious or spiritual beliefs.

S13) The religious or spiritual beliefs of my family member.

**Patient-centric, life-long narrative**

A4) What promotes the life patient has valued.

N4) What promotes the life the patient has valued.

P4) What promotes the life you have valued.

S4) What promotes the life my family member has valued.

A6) The way patient used to make decisions.

N6) The way the patient used to make decisions.

P6) The way you used to make decisions.

S6) The way my family member used to make decisions.

A2) What continues the life patient has led.

N2) What continues the life the patient has led.

P2) What continues the life you have led.

S2) What continues the life my family member has led.

A18) Life-long story of patient.

N18) The life-long story of the patient.

P18) Your life-long story.

S18) The life-long story of my family member.

**Patient-centric, health**

A21) Improving patient health.

N21) Improving the patient’s health.

P21) Improving your health.

S21) Improving my family member’s health.

A28) What is in the best interests of patient.

N28) What is in the best interests of the patient.

P28) What is in the best interests of you.

S28) What is in the best interests of my family member.

A12) Patient pain and suffering.

N12) Patient’s pain and suffering.

P12) Your pain and suffering.

S12) My family member’s pain and suffering.

A22) Medical facts.

N22) The medical facts, such as patient’s condition, the risks and benefits of the procedures, and possible outcomes for the patient.

P22) The medical facts, such as your condition, the risks and benefits of the procedures, and possible outcomes for you.

S22) The medical facts, such as my family member’s condition, the risks and benefits of the procedures, and my family member’s possible outcomes.

A3) Trying everything possible to save patient.**^2^**

N3) Trying everything possible to save the patient.

P3) Trying everything possible to save you.

S3) Trying everything possible to save family member.

**Surrogate-centric, emotion**

A26) Fear of loss of loved one.

N26) Fear of loss of a loved one.

P26) Fear of loss of you.

S26) Fear of loss of my loved family member.

A25) Feeling of guilt because not trying everything possible.

N25) Feeling of guilt because not trying everything possible.

P25) Feeling of guilt because not trying everything possible.

S25) Feeling of guilt because not trying everything possible.

**Surrogate-centric, preference**

A8) What surrogate would have wanted if in similar situation.

N8) What the surrogate (the family member who is making the decision for the patient) would have wanted if he/she were in a similar situation.

P8) What the surrogate (the family member who is making the decision for you) would have wanted if he/she were in a similar situation.

S8) What I would have wanted if I were in a similar situation.

A10) Surrogate own wishes for patient.

N10) The surrogate’s (the family member who is making the decision for the patient) own wishes for the patient.

P10) The surrogate’s (the family member who is making the decision for you) own wishes for you.

S10) My own wishes for my family member.

A7) Surrogate own religious or spiritual beliefs.

N7) The surrogate’s (the family member who is making the decision for the patient) own religious or spiritual beliefs.

P7) The surrogate’s (the family member who is making the decision for you) own religious or spiritual beliefs.

S7) My own religious or spiritual beliefs.

**Surrogate-centric, interest**

A14) Surrogate burden due to patient care or disliked outcome.

N14) The burden on the surrogate (the family member who is making the decision for the patient) in regard to taking care of the patient or as a result of disliked outcome.

P14) The burden on the surrogate (the family member who is making the decision for you) in regard to taking care of you or as a result of disliked outcome.

S14) The burden on me in regard to taking care of my family member or as a result of a disliked outcome.

A23) Surrogate needs.

N23) The surrogate’s (the family member who is making the decision for the patient) needs.

P23) The surrogate’s (the family member who is making the decision for you) needs.

S23) My own needs.

A17) Financial concerns. **^3^**

N17) Concerns about insurance, finances, or paying for medical care.

P17) Concerns about insurance, finances, or paying for medical care.

S17) Concerns about insurance, finances, or paying for medical care.

**Family-centric**

A9) Family needs.

N9) The needs of the family.

P9) The needs of your family.

S9) The needs of my family.

A20) Reaching family agreement to maintain family cohesion.

N20) Reaching an agreement about the decision with family members to maintain family cohesion.

P20) Reaching an agreement about the decision with other members of your family to maintain family cohesion.

S20) Reaching an agreement about the decision with my other family members to maintain family cohesion.

A19) Reaching family agreement to distribute responsibility.

N19) Reaching an agreement about the decision with family members to distribute responsibility for the decision.

P19) Reaching an agreement about the decision with other members of your family to distribute responsibility for the decision.

S19) Reaching an agreement about the decision with my other family members to distribute responsibility for the decision.

A27) Family burden due to patient care or as disliked outcome.

N27) The burden on the family in regard to taking care of the patient or as a result of a disliked outcome.

P27) The burden on your family in regard to taking care of you or as a result of disliked outcome.

S27) The burden on my family in regard to taking care of my family member or as a result of disliked outcome.

**Society-centric**

A1) Effect of caring for patient on patients with same disease.

N1) The effect of caring for the patient on patients with the same disease.

P1) The effect of caring for you on patients with the same disease.

S1) The effect of caring for my family member on patients with the same disease.

A5) Effect of caring for patient on all patients in society.

N5) The effect of caring for the patient on all other patients in the society.

P5) The effect of caring for you on all other patients in the society.

S5) The effect of caring for my family member on all other patients in the society.

A16) Cost to society from caring for patient.

N16) The cost to society from caring for the patient.

P16) The cost to society from caring for you.

S16) The cost to society from caring for my family member.

^1^ Can also be classified under patient-centric, life-long narrative. ^2^ Can also be classified under surrogate-centric, emotion. ^3^ Can also be classified under family-centric.
