# Supplementary material for: Factors underlying surrogate medical decision-making in middle eastern and east Asian women: a Q-methodology study
Source: BMC Palliat Care. 2020 Sep 1;19:137. doi: 10.1186/s12904-020-00643-9 (PMC7466416; doi:10.1186/s12904-020-00643-9)
Supplement: Supplementary file 2 — Additional file 2. Instructions and sorting Sheet. [file 12904_2020_643_MOESM2_ESM.docx]

**Additional file 2, Instructions and sorting Sheet**

**1. Instructions to sorters**

**1.1 Instructions for Q-sorting according to norm-perception (N-perspective)**

The attached 28 items represent potential factors or principles that may be considered when a family member makes a medical decision on behalf of an adult relative who cannot make, or cannot participate in, medical decisions due to mental impairment. Such decisions may cover procedures and surgeries, life sustaining care (such as code status, intubation, artificial nutrition), and hospital discharge to a nursing home or similar institutions. This is called surrogate decision-making. Please sort the 28 items by ranking them from 1 to 9 using the attached sorting sheet. Please note the following:

-Rank 1 should be assigned to the factors/principle that you think should be considered the least important (or not important at all) in surrogate decision-making, in general and regardless of your own preferences.

-Rank 9 should be assigned to the factor/principle that you think should be considered the most important in surrogate decision-making, again in general and regardless of your own preferences.

-The statements are presented in a random order.

**1.2 Instructions for Q-sorting according to patient’s perspective (P-perspective)**

**Preferred way of having surrogate-decision made on my behalf**

The attached 28 items represent potential factors or principles that may be considered when a family member makes a medical decision on behalf of an adult relative who cannot make, or cannot participate in, medical decisions due to mental impairment. Such decisions may cover procedures and surgeries, life sustaining care (such as code status, intubation, artificial nutrition), and hospital discharge to a nursing home or similar institutions. This is called surrogate decision-making. Please sort the 28 items by ranking them from 1 to 9 using the attached sorting sheet. Please note the following:

-Rank 1 should be assigned to the factor/principle that you personally prefer to be considered the least important (or not important at all) when a family member makes a decision on your behalf.

-Rank 9 should be assigned to the factor/principle that you personally prefer to be considered the most important when a family member makes a decision on your behalf.

-The statements are presented in a random order.

**1.3 Instructions for Q-sorting according to surrogate’s perspective (S-perspective)**

**Preferred way of making surrogate decisions for my family**

The attached 28 items represent potential factors or principles that may be considered when a family member makes a medical decision on behalf of an adult relative who cannot make, or cannot participate in, medical decisions due to mental impairment. Such decisions may cover procedures and surgeries, life sustaining care (such as code status, intubation, artificial nutrition), and hospital discharge to a nursing home or similar institutions. This is called surrogate decision-making. Please sort the 28 items by ranking them from 1 to 9 using the attached sorting sheet. Please note the following:

-Rank 1 should be assigned to the factor/principle that you personally prefer to consider the least important (or not important at all) when you make a decision on behalf of a member of your family.

-Rank 9 should be assigned to the factor/principle that you personally prefer to consider the most important when you make a decision on behalf of a member of your family.

-The statements are presented in a random order.

**1.4 General instructions**

Please read the following instructions carefully before you start your sort. It is very important that the sorting procedure be followed in all its details.

Just to be clear, we are interested in your own point of view. Therefore, there is no right or wrong answer.

Your response will be treated as highly confidential and will be used only for the purpose of this study. It will not be linked to your (or your family) medical records or used to inform your medical management.

Read through all the items carefully to get a general impression of the range of issues at hand. First, divide the items into three piles, most important, least important, and “others”. Then look through the most important pile and pick out the one item that is most important. Place it in the box under number “9” in the Q-sort grid that is given to you. Now, look through the same pile again and pick out the two items that you consider most important (excluding from consideration the one you have already put under number “9”). Place them in the two boxes under number “8” in the Q-sort grid. It does not matter which one goes on top or in the bottom. Now of those items that remain in the most important pile, pick out three items that you consider most important (excluding from consideration those items you have already put under numbers “9” and “8”). Place them in the three boxes under number “7” in the Q-sort grid. Again, it does not matter which one goes on top or in the bottom.

Next, work from the opposite end toward the middle. Of those items in the least important pile, pick out the one item that you consider least important. Place it in the box under number “1” in the Q-sort grid. Now look through the same pile again and pick out the two items that you consider least important (excluding from consideration the one that you have already put under number “1”). Place them in the two boxes under number “2” in the Q-sort grid. Now of those items that remain in the least important pile, choose the three items that you consider least important. Place them in the three boxes under number “3” in the Q-sort grid. Again, it does not matter which one goes on top or in the bottom.

Now you have to sort out the remaining 16 items in the “others” pile. Pick up the 5 items that you consider most important. Place them in the five boxes under number “6” in the Q-sort grid. Pick up the 5 items that you consider least important. Place them in the five boxes under number “4” in the Q-sort grid. Pick up the 6 items that are left in the pile, place them in the six boxes under number “5” in the Q-sort grid. Again, it does not matter which one goes on top or in the bottom.

You may have difficulty placing the required number of items into each of the 9 categories. For example, if 3 items are required for a category, you may find that you have too many or too few. In either event, finish with the required number of items, either by eliminating those that can most sensibly be moved out or by moving in those items that are most relevant. You may feel that some of your placements are forced. Your task may be admittedly an awkward one, but try to work through it anyway.

Before finalizing your Q-sort, make sure your opinion is reflected on the Q-sort grid in front of you. Feel free to rearrange any/all items, so that when you are done, the positions of the items relative to each other reflect how you feel, as closely as it can be. When you are done, please write down the number of each card in the boxes of the Q-sorting sheet that correspond to the boxes of the Q-sort grid that you have placed the cards in. The Q-sorting sheet has the same arrangement as the Q-sort grid. We will collect your Q-sorting sheet to analyze it. After writing the numbers of all the cards in the corresponding boxes of the Q-sorting sheet, please explain/give us your comments why the three items you have placed under number “ 8 and 9” are the most important and why the three items you have placed under number “1 and 2” are the least important (please use the attached sheet). This will help us understand your opinion.

We would like to emphasize that the worth of this research is heavily dependent on how well and conscientiously participating people perform their tasks. Sorting the items as described above is perhaps tedious. But when honestly done, the results would be very useful. On the other hand, analysis of items that have been haphazardly positioned or positioned without due considerations would lead to wrong conclusions. Therefore, we would like to request that you return the material to the study coordinator without your response, if for any reason you feel that you cannot, or prefer not to, perform the task in a meaningful manner.

Thank you for your cooperation.

**2. Sorting sheet**

| **Most important 9** | **8** | **7** | **6** | **5** | **4** | **3** | **2** | **Least important 1** |
| --- | --- | --- | --- | --- | --- | --- | --- | --- |
|  |  |  |  |  |  |  |  |  |
|  |  |  |  |  |  |  |  |  |
|  |  |  |  |  |  |  |  |  |
|  |  |  |  |  |  |  |  |  |
|  |  |  |  |  |  |  |  |  |
|  |  |  |  |  |  |  |  |  |

Please explain why the three items you have placed below the “8 and 9” are most important.

Card #: ______________________________________________________________________________

______________________________________________________________________________Card #:

______________________________________________________________________________

______________________________________________________________________________

Card #:

______________________________________________________________________________

______________________________________________________________________________

Please explain why you the three items you have placed below the “1 and 2” are least important.

Card #

______________________________________________________________________________

______________________________________________________________________________

Card #

____________________________________________________________________________________________________________________________________________________________

Card #

____________________________________________________________________________________________________________________________________________________________
