# Supplementary material for: Factors underlying surrogate medical decision-making in middle eastern and east Asian women: a Q-methodology study
Source: BMC Palliat Care. 2020 Sep 1;19:137. doi: 10.1186/s12904-020-00643-9 (PMC7466416; doi:10.1186/s12904-020-00643-9)
Supplement: Supplementary file 3 — Additional file 3. Social value scale. [file 12904_2020_643_MOESM3_ESM.docx]

| **Additional file 3-social value scale**  **Table 1S:** Social value scale scores of 120 Middle Eastern and 120 East Asian women | | |
| --- | --- | --- |
|  | **Middle Eastern** | **East**  **Asian** |
| **Sub-scale-1: Criteria for planning social change** | | |
| 1. Despite the rapid pace of technological change, it is preferable that social change (family, society) progress in a conservative manner (C) | 3.7 (1.2) | 3.9 (0.9) |
| 2. Many segments of society live in distress and poverty, and it is important that the government itself takes responsibility to rapidly execute social change in order to help them (L) | 4.2 (1.1) | 4.2 (1.0) |
| 3. It is important that the status of women change in accord with progressive social change (L) | 4.0 (1.1) | 3.9 (1.1) |
| 4. Traditional precedents should play an important role in planning social changes (C) | 3.2 (1.3) | 3.8 (1.0) |
| 5. Social research and progress in the social sciences are the most constructive and meaningful basis for planning successful social change (L) | 4.5 (0.8) | 4.4 (0.8) |
| 6. Change in the status of women should be not only in accord with social change, but it is important to give weight to traditional precedents (C) | 3.9 (1.2) | 4.1 (0.9) |
| **Sub-scale-2: Individual free choice with regard to medical ethical questions** | | |
| 1. The individual conscience and the right to self-determination should be the highest authority and the final word with regard to questions of medical ethics concerning an individual’s own body (L) | 3.9 (1.3) | 4.2 (1.0) |
| 2. The proprietorship on individual life is shared between man and God (C) | 4.5 (0.9) | 4.5 (0.8) |
| 3. The individual is the sole proprietor on his body (L) | 3.5 (1.6) | 4.3 (1.1) |
| 4. Medical ethical decisions should take into account the welfare of the individual patients, but also be coordinated with precedents of religious law (C) | 4.1 (1.1) | 4.0 (0.9) |
| 5. With regard to medical ethical questions, in my own personal life, I would not make a decision without consulting a religious leader (C) | 3.4 (1.3) | 3.0 (1.3) |
| 6.With regard to medical ethical questions, it is important that the individual see himself as a link in a collective tradition (nation, religion, or ethnic group) (C) | 3.4 (1.3) | 3.4 (1.2) |
| 7. It is important that the individual have full freedom to fulfill his life in accord with his personal philosophy (L) | 3.5 (1.2) | 4.3 (0.9) |
| **Subscale-3: Criteria for deciding the ‘Good’ and the ‘Bad’ with regard to the personal welfare of the individual** | | |
| 1. There exists a spiritual reality that is truer in its essence than the material physical reality that we are able to know through our senses and reason (C) | 4.0 (1.0) | 4.0 (1.0) |
| 2. We must recognize and know the reality and essence of our existence primarily through the use of our reason in a scientific and rational manner (L) | 4.2 (0.9) | 4.0 (0.9) |
| 3. Every individual has a soul that is derived from an eternal spiritual world (C) | 3.7 (1.3) | 4.1 (1.0) |
| 4. Even if there is some type of spiritual reality, it cannot be an ultimate authority for deciding what is ‘good’ and ‘bad’, and what is ‘true’ & ‘not true’ (L) | 3.4 (1.3) | 3.8 (1.1) |
| 5. The most meaningful basis for deciding ‘good’ and ‘bad’, ‘true and not true’ is divine revelation (C) | 4.0 (1.2) | 3.8 (1.1) |
| 6. In understanding what constitutes ‘personal welfare’, we want to focus on the person’s emotional and material needs because it is not possible with any degree of truthfulness to know what constitutes the spiritual welfare of the individual (L) | 3.9 (1.1) | 3.8 (0.9) |

Data are mean (SD) on 5-point scale (from 1=total disagreement to 5=full agreement). C, “conservative”; L, “liberal”.
