# Supplementary material for: Factors underlying surrogate medical decision-making in middle eastern and east Asian women: a Q-methodology study
Source: BMC Palliat Care. 2020 Sep 1;19:137. doi: 10.1186/s12904-020-00643-9 (PMC7466416; doi:10.1186/s12904-020-00643-9)
Supplement: Supplementary file 4 — Additional file 4. factor characteristics and item scores. [file 12904_2020_643_MOESM4_ESM.docx]

**Additional file 4-factor characteristics and item scores**

**Table 2S:** Factor characteristics and item scores of six surrogate’s decision-making Q methodology models of Middle Eastern women according to norm-perception (N-perspective)

**Table 3S:** Factor characteristics and item scores of six surrogate’s decision-making Q-methodology models of Middle Eastern women according to patient’s perspective (P-perspective)

**Table 4S:** Factor characteristics and item scores of six surrogate’s decision-making Q-methodology models of Middle Eastern women according to surrogate’s perspective (S-perspective)

**Table 5S:** Factor characteristics and item scores of six surrogate’s decision-making Q-methodology models of East Asian women according to norm-perception (N-perspective)

**Table 6S:** Factor characteristics and item scores of six surrogate’s decision-making Q-methodology models of East Asian women according to patient’s perspective (P-perspective)

**Table 7S:** Factor characteristics and item scores of six surrogate’s decision-making Q-methodology models of East Asian women according to surrogate’s perspective (S-perspective)

**Table 2S:** Factor characteristics and item scores of six surrogate’s decision-making Q-methodology models of Middle Eastern women according to norm-perception (N-perspective)

|  | | **Model** | | | | | |
| --- | --- | --- | --- | --- | --- | --- | --- |
|  | | **A** | **B** | **C** | **D** | **E** | **F** |
| **Number of defining Q-sorts** | | 19 | 13 | 14 | 10 | 12 | 10 |
| **Variance explained (%)** | | 13 | 9 | 8 | 10 | 10 | 8 |
| **Eigenvalue** | | 15.9 | 11.0 | 9.7 | 12.0 | 12.3 | 9.4 |
| **Items and scores** | | | | | | | |
| 1n | Effect of caring for patient on patients with same disease. | 4 | 6 | 6 | 4 | 3 | 2 |
| 2n | What continues the life patient has led. | 7 | 6 | 4 | 5 | 5 | 4 |
| 3 | Trying everything possible to save patient.* | 9 | 8 | 9 | 9 | 8 | 9 |
| 4n | What promotes the life patient has valued. | 5 | 6 | 4 | 3 | 6 | 5 |
| 5n | Effect of caring for patient on all patients in society. | 3 | 5 | 5 | 3 | 3 | 4 |
| 6n | The way patient used to make decisions. | 5 | 5 | 3 | 5 | 6 | 6 |
| 7n | Surrogate own religious or spiritual beliefs. | 5 | 1 | 6 | 2 | 5 | 3 |
| 8n | What surrogate would have wanted if in similar situation. | 4 | 5 | 5 | 3 | 4 | 5 |
| 9n | Family needs. | 3 | 3 | 2 | 6 | 2 | 5 |
| 10n | Surrogate own wishes for patient. | 5 | 2 | 5 | 4 | 5 | 5 |
| 11n | What patient requested in advance directives. | 6 | 6 | 4 | 5 | 8 | 7 |
| 12 | Patient pain and suffering. | 7 | 7 | 7 | 7 | 9 | 8 |
| 13n | Religious or spiritual beliefs of patient. | 4 | 4 | 7 | 5 | 6 | 3 |
| 14n | Surrogate burden due to patient care or disliked outcome. | 2 | 3 | 6 | 6 | 4 | 5 |
| 15n | What patient would have wanted based on past wishes.* | 6 | 5 | 5 | 6 | 6 | 6 |
| 16n | Cost to society from caring for patient. | 4 | 7 | 3 | 2 | 2 | 1 |
| 17n | Financial concerns. | 1 | 6 | 6 | 6 | 3 | 2 |
| 18 | Life-long story of patient. | 5 | 3 | 1 | 1 | 5 | 4 |
| 19n | Reaching family agreement to distribute responsibility. | 6 | 5 | 4 | 5 | 4 | 6 |
| 20n | Reaching family agreement to maintain family cohesion. | 5 | 4 | 3 | 4 | 4 | 6 |
| 21 | Improving patient health.* | 8 | 7 | 8 | 8 | 7 | 7 |
| 22 | Medical facts. | 7 | 9 | 8 | 6 | 7 | 8 |
| 23 | Surrogate needs. | 4 | 2 | 5 | 4 | 1 | 4 |
| 24n | What patient wants now despite mental impairment. | 6 | 4 | 5 | 5 | 5 | 4 |
| 25n | Feeling of guilt because not trying everything possible. | 2 | 4 | 4 | 7 | 6 | 5 |
| 26n | Fear of loss of loved one. | 6 | 4 | 2 | 7 | 5 | 3 |
| 27n | Family burden due to patient care or disliked outcome. | 3 | 5 | 6 | 4 | 4 | 6 |
| 28 | What is in the best interests of patient.* | 8 | 8 | 7 | 8 | 7 | 7 |

The six-factor solution accounted for 58% of the total variance and 78 (65%) of 120 Q-sorts (35 Q-sorts did not have significant loading and 7 were confounded). Item scores represent idealized scores of the 28 items for each program-generated model. Items numbers and abbreviated version are presented; full version is available in Additional file 1-Q-set items. “n” denotes neutral item on averaging-analysis (i.e., mean ranking score ˃4 and <6). “*” denotes consensus item on Q-methodology analysis. There were no differentiating items. Item scores correlation ranged from 0.32 (models A and C) to 0.69 (models E and F).

**Table 3S:** Factor characteristics and item scores of six surrogate’s decision-making Q-methodology models of Middle Eastern women according to patient’s perspective (P-perspective)

|  | | **Model** | | | | | |
| --- | --- | --- | --- | --- | --- | --- | --- |
|  | | **A** | **B** | **C** | **D** | **E** | **F** |
| **Number of defining Q-sorts** | | 14 | 11 | 15 | 9 | 11 | 16 |
| **Variance explained (%)** | | 10 | 8 | 11 | 7 | 9 | 11 |
| **Eigenvalue** | | 12.5 | 9.8 | 13.7 | 8.3 | 10.8 | 12.7 |
| **Items and scores** | | | | | | | |
| 1n | Effect of caring for patient on patients with same disease.* | 3 | 3 | 3 | 4 | 3 | 3 |
| 2n | What continues the life patient has led. | 3 | 4 | 5 | 3 | 5 | 6 |
| 3 | Trying everything possible to save patient. | 8 | 8 | 9 | 7 | 8 | 7 |
| 4n | What promotes the life patient has valued. | 4 | 3 | 6 | 3 | 4 | 6 |
| 5n | Effect of caring for patient on all patients in society. | 2 | 2 | 2 | 4 | 3 | 2 |
| 6n | The way patient used to make decisions. | 4 | 6 | 6 | 4 | 5 | 6 |
| 7n | Surrogate own religious or spiritual beliefs. | 5 | 4 | 3 | 2 | 5 | 2 |
| 8n | What surrogate would have wanted if in similar situation. | 5 | 4 | 6 | 6 | 6 | 5 |
| 9n | Family needs. | 6 | 7 | 4 | 8 | 6 | 4 |
| 10n | Surrogate own wishes for patient. | 5 | 4 | 6 | 5 | 5 | 4 |
| 11n | What patient requested in advance directives. | 4 | 6 | 5 | 5 | 4 | 9 |
| 12 | Patient pain and suffering. | 9 | 8 | 8 | 6 | 7 | 6 |
| 13n | Religious or spiritual beliefs of patient. | 7 | 7 | 4 | 1 | 6 | 5 |
| 14n | Surrogate burden due to patient care or disliked outcome. | 6 | 5 | 4 | 6 | 5 | 5 |
| 15n | What patient would have wanted based on past wishes. | 6 | 5 | 6 | 5 | 5 | 8 |
| 16 | Cost to society from caring for patient. | 5 | 2 | 1 | 4 | 2 | 1 |
| 17n | Financial concerns. | 7 | 1 | 2 | 5 | 4 | 3 |
| 18 | Life-long story of patient. | 1 | 4 | 4 | 5 | 2 | 4 |
| 19n | Reaching family agreement to distribute responsibility. | 5 | 5 | 5 | 6 | 7 | 5 |
| 20n | Reaching family agreement to maintain family cohesion. | 5 | 5 | 5 | 7 | 7 | 5 |
| 21 | Improving patient health. | 8 | 7 | 7 | 9 | 9 | 7 |
| 22 | Medical facts. | 7 | 6 | 7 | 7 | 8 | 8 |
| 23n | Surrogate needs. | 3 | 3 | 4 | 5 | 6 | 3 |
| 24n | What patient wants now despite mental impairment. | 4 | 5 | 5 | 4 | 4 | 6 |
| 25n | Feeling of guilt because not trying everything possible. | 4 | 6 | 5 | 2 | 1 | 4 |
| 26n | Fear of loss of loved one. | 2 | 6 | 8 | 3 | 3 | 4 |
| 27n | Family burden due to patient care or disliked outcome. | 6 | 5 | 3 | 6 | 4 | 5 |
| 28 | What is in the best interests of patient. | 6 | 9 | 7 | 8 | 6 | 7 |

The six-factor solution accounted for 56% of the total variance and 76 (63%) of 120 Q-sorts (40 Q-sorts did not have significant loading and 4 were confounded). Item scores represent idealized scores of the 28 items for each program-generated model. Items numbers and abbreviated version are presented; full version is available in Additional file 1-Q-set items. “n” denotes neutral item on averaging-analysis (i.e., mean ranking score ˃4 and <6). “*” denotes consensus item on Q-methodology analysis. There were no differentiating items. Item scores correlation ranged from 0.32 (models A and C) to 0.69 (models C and F).

**Table 4S:** Factor characteristics and item scores of six surrogate’s decision-making Q-methodology models of Middle Eastern women according to surrogate’s perspective (S-perspective)

|  | | **Model** | | | | | |
| --- | --- | --- | --- | --- | --- | --- | --- |
|  | | **A** | **B** | **C** | **D** | **E** | **F** |
| **Number of defining Q-sorts** | | 14 | 21 | 8 | 9 | 20 | 12 |
| **Variance explained (%)** | | 11 | 13 | 8 | 8 | 13 | 11 |
| **Eigenvalue** | | 13.1 | 15.1 | 9.2 | 9.2 | 15.3 | 13.6 |
| **Items and scores** | | | | | | | |
| 1n | Effect of caring for patient on patients with same disease.* | 3 | 3 | 3 | 3 | 4 | 3 |
| 2n | What continues the life patient has led. | 6 | 4 | 4 | 4 | 5 | 5 |
| 3 | Trying everything possible to save patient. | 6 | 8 | 8 | 9 | 9 | 9 |
| 4n | What promotes the life patient has valued. | 6 | 5 | 3 | 4 | 6 | 5 |
| 5 | Effect of caring for patient on all patients in society. | 3 | 3 | 1 | 3 | 4 | 3 |
| 6n | The way patient used to make decisions. | 6 | 4 | 4 | 2 | 5 | 6 |
| 7n | Surrogate own religious or spiritual beliefs. | 1 | 7 | 2 | 5 | 3 | 6 |
| 8n | What surrogate would have wanted if in similar situation.* | 5 | 6 | 5 | 5 | 6 | 6 |
| 9n | Family needs. | 3 | 4 | 7 | 3 | 2 | 4 |
| 10n | Surrogate own wishes for patient. | 4 | 6 | 5 | 4 | 7 | 5 |
| 11n | What patient requested in advance directives. | 9 | 5 | 4 | 6 | 5 | 5 |
| 12 | Patient pain and suffering. | 7 | 7 | 7 | 8 | 6 | 7 |
| 13n | Religious or spiritual beliefs of patient. | 5 | 6 | 2 | 7 | 2 | 6 |
| 14n | Surrogate burden due to patient care or disliked outcome. | 5 | 4 | 6 | 5 | 5 | 4 |
| 15n | What patient would have wanted based on past wishes. | 8 | 5 | 6 | 5 | 5 | 5 |
| 16 | Cost to society from caring for patient. | 2 | 2 | 3 | 4 | 4 | 1 |
| 17n | Financial concerns. | 4 | 2 | 5 | 7 | 4 | 2 |
| 18 | Life-long story of patient. | 5 | 4 | 5 | 1 | 3 | 2 |
| 19n | Reaching family agreement to distribute responsibility. | 4 | 5 | 6 | 5 | 6 | 7 |
| 20n | Reaching family agreement to maintain family cohesion. | 4 | 5 | 7 | 5 | 6 | 6 |
| 21 | Improving patient health.* | 7 | 8 | 8 | 8 | 7 | 8 |
| 22 | Medical facts. | 8 | 6 | 5 | 7 | 8 | 8 |
| 23 | Surrogate needs. | 2 | 1 | 4 | 2 | 1 | 3 |
| 24n | What patient wants now despite mental impairment. | 6 | 5 | 4 | 4 | 5 | 4 |
| 25n | Feeling of guilt because not trying everything possible. | 5 | 7 | 5 | 6 | 3 | 4 |
| 26n | Fear of loss of loved one. | 5 | 9 | 9 | 6 | 7 | 4 |
| 27n | Family burden due to patient care or disliked outcome. | 4 | 3 | 6 | 6 | 4 | 5 |
| 28 | What is in the best interests of patient. | 7 | 6 | 6 | 6 | 8 | 7 |

The six-factor solution accounted for 64% of the total variance and 74 (62%) of 120 Q-sorts (23 Q-sorts did not have significant loading and 13 were confounded). Item scores represent idealized scores of the 28 items for each program-generated model. Items numbers and abbreviated version are presented; full version is available in Additional file 1-Q-set items. “n” denotes neutral item on averaging-analysis (i.e., mean ranking score ˃4 and <6). “*” denotes consensus item on Q-methodology analysis. There were no differentiating items. Item scores correlation ranged from 0.33 (models A and C) to 0.68 (models B and F).

**Table 5S:** Factor characteristics and item scores of six surrogate’s decision-making Q-methodology models of East Asian women according to norm-perception (N-perspective)

|  | | **Model** | | | | | |
| --- | --- | --- | --- | --- | --- | --- | --- |
|  | | **A** | **B** | **C** | **D** | **E** | **F** |
| **Number of defining Q-sorts** | | 12 | 8 | 13 | 18 | 16 | 9 |
| **Variance explained (%)** | | 9 | 9 | 9 | 10 | 10 | 7 |
| **Eigenvalue** | | 10.6 | 11.0 | 10.4 | 12.3 | 11.7 | 8.9 |
| **Items and scores** | | | | | | | |
| 1n | Effect of caring for patient on patients with same disease. | 3 | 4 | 4 | 3 | 4 | 5 |
| 2n | What continues the life patient has led. | 4 | 5 | 3 | 4 | 4 | 6 |
| 3 | Trying everything possible to save patient. | 8 | 7 | 9 | 9 | 6 | 7 |
| 4n | What promotes the life patient has valued. | 5 | 5 | 4 | 6 | 5 | 6 |
| 5n | Effect of caring for patient on all patients in society. | 2 | 4 | 4 | 3 | 3 | 4 |
| 6n | The way patient used to make decisions. | 4 | 6 | 5 | 6 | 5 | 4 |
| 7n | Surrogate own religious or spiritual beliefs. | 6 | 1 | 2 | 6 | 4 | 5 |
| 8n | What surrogate would have wanted if in similar situation.* | 4 | 4 | 5 | 4 | 4 | 5 |
| 9n | Family needs. | 5 | 6 | 4 | 5 | 5 | 6 |
| 10n | Surrogate own wishes for patient. | 3 | 3 | 5 | 4 | 2 | 2 |
| 11n | What patient requested in advance directives. | 7 | 6 | 3 | 6 | 8 | 6 |
| 12n | Patient pain and suffering. | 9 | 7 | 7 | 7 | 6 | 7 |
| 13n | Religious or spiritual beliefs of patient. | 7 | 2 | 3 | 7 | 7 | 8 |
| 14n | Surrogate burden due to patient care or disliked outcome. | 2 | 3 | 6 | 4 | 4 | 2 |
| 15n | What patient would have wanted based on past wishes. | 6 | 5 | 4 | 5 | 6 | 4 |
| 16 | Cost to society from caring for patient. | 1 | 3 | 2 | 1 | 3 | 3 |
| 17n | Financial concerns. | 6 | 5 | 5 | 2 | 6 | 6 |
| 18 | Life-long story of patient. | 4 | 5 | 1 | 3 | 2 | 1 |
| 19n | Reaching family agreement to distribute responsibility. | 5 | 6 | 7 | 4 | 5 | 4 |
| 20n | Reaching family agreement to maintain family cohesion.* | 5 | 6 | 6 | 5 | 6 | 5 |
| 21 | Improving patient health.* | 8 | 9 | 8 | 8 | 8 | 9 |
| 22n | Medical facts. | 7 | 7 | 8 | 5 | 7 | 5 |
| 23n | Surrogate needs. | 3 | 2 | 6 | 5 | 5 | 4 |
| 24n | What patient wants now despite mental impairment. | 5 | 4 | 5 | 6 | 7 | 5 |
| 25n | Feeling of guilt because not trying everything possible. | 6 | 5 | 6 | 5 | 3 | 3 |
| 26n | Fear of loss of loved one.** | 6 | 8 | 6 | 7 | 1 | 7 |
| 27n | Family burden due to patient care or disliked outcome. | 4 | 4 | 5 | 2 | 5 | 3 |
| 28 | What is in the best interests of patient. | 5 | 8 | 7 | 8 | 9 | 8 |

The six-factor solution accounted for 54% of the total variance and 76 (63%) of 120 Q-sorts (42 Q-sorts did not have significant loading and 2 were confounded). Item scores represent idealized scores of the 28 items for each program-generated model. Items numbers and abbreviated version are presented; full version is available in Additional file 1-Q-set items. “n” denotes neutral item on averaging-analysis (i.e., mean ranking score ˃4 and <6). “*” denotes consensus item and “**” denotes differentiating item on Q-methodology analysis. Item scores correlation ranged from 0.34 (models B and E; models C and F) to 0.69 (models D and F).

**Table 6S:** Factor characteristics and item scores of six surrogate’s decision-making Q-methodology models of East Asian women according to patient’s perspective (P-perspective)

|  | | **Resolution model** | | | | | |
| --- | --- | --- | --- | --- | --- | --- | --- |
|  | | **A** | **B** | **C** | **D** | **E** | **F** |
| **Number of defining Q-sorts** | | 16 | 12 | 11 | 8 | 10 | 8 |
| **Variance explained (%)** | | 12 | 9 | 9 | 7 | 7 | 8 |
| **Eigenvalue** | | 13.9 | 10.2 | 10.3 | 8.9 | 8.3 | 9.8 |
| **Items and scores** | | | | | | | |
| 1n | Effect of caring for patient on patients with same disease. | 2 | 5 | 2 | 4 | 2 | 4 |
| 2n | What continues the life patient has led. | 5 | 6 | 4 | 3 | 4 | 5 |
| 3 | Trying everything possible to save patient. | 9 | 8 | 6 | 5 | 8 | 6 |
| 4n | What promotes the life patient has valued. | 5 | 6 | 4 | 6 | 3 | 6 |
| 5n | Effect of caring for patient on all patients in society. | 2 | 4 | 3 | 4 | 4 | 6 |
| 6n | The way patient used to make decisions. | 6 | 6 | 6 | 5 | 5 | 2 |
| 7n | Surrogate own religious or spiritual beliefs. | 5 | 2 | 4 | 7 | 5 | 4 |
| 8n | What surrogate would have wanted if in similar situation. | 4 | 3 | 5 | 3 | 6 | 4 |
| 9 | Family needs. | 6 | 7 | 7 | 8 | 6 | 7 |
| 10n | Surrogate own wishes for patient. | 3 | 4 | 4 | 5 | 5 | 5 |
| 11n | What patient requested in advance directives. | 7 | 5 | 7 | 5 | 4 | 2 |
| 12n | Patient pain and suffering. | 8 | 3 | 7 | 6 | 3 | 8 |
| 13n | Religious or spiritual beliefs of patient. | 7 | 1 | 3 | 9 | 8 | 6 |
| 14n | Surrogate burden due to patient care or disliked outcome. | 3 | 4 | 5 | 5 | 7 | 4 |
| 15n | What patient would have wanted based on past wishes. | 5 | 7 | 5 | 4 | 2 | 5 |
| 16 | Cost to society from caring for patient | 1 | 5 | 4 | 4 | 3 | 3 |
| 17n | Financial concerns. | 4 | 7 | 9 | 7 | 7 | 5 |
| 18 | Life-long story of patient. | 5 | 2 | 1 | 1 | 1 | 1 |
| 19n | Reaching family agreement to distribute responsibility.* | 6 | 5 | 5 | 6 | 5 | 5 |
| 20n | Reaching family agreement to maintain family cohesion.* | 6 | 6 | 5 | 6 | 6 | 5 |
| 21 | Improving patient health.* | 8 | 9 | 8 | 8 | 9 | 9 |
| 22n | Medical facts. | 4 | 8 | 8 | 7 | 5 | 8 |
| 23n | Surrogate needs. | 4 | 4 | 6 | 5 | 4 | 7 |
| 24n | What patient wants now despite mental impairment. | 6 | 5 | 5 | 4 | 5 | 4 |
| 25n | Feeling of guilt because not trying everything possible. | 3 | 3 | 3 | 2 | 6 | 3 |
| 26n | Fear of loss of loved one. | 5 | 5 | 2 | 2 | 6 | 7 |
| 27n | Family burden due to patient care or disliked outcome. | 4 | 4 | 6 | 6 | 7 | 6 |
| 28n | What is in the best interests of patient. | 7 | 6 | 6 | 3 | 4 | 3 |

The six-factor solution accounted for 52% of the total variance and 65 (54%) of 120 Q-sorts (51 Q-sorts did not have significant loading and 4 were confounded). Item scores represent idealized scores of the 28 items for each program-generated model. Items numbers and abbreviated version are presented; full version is available in Additional file 1-Q-set items. “n” denotes neutral item on averaging-analysis (i.e., mean ranking score ˃4 and <6). “*” denotes consensus item on Q-methodology analysis. There were no differentiating items. Item scores correlation ranged from 0.20 (models B and E) to 0.58 (models B and C).

**Table 7S:** Factor characteristics and item scores of six surrogate’s decision-making Q-methodology models of East Asian women according to surrogate’s perspective (S-perspective)

|  | | **Model** | | | | | |
| --- | --- | --- | --- | --- | --- | --- | --- |
|  | | **A** | **B** | **C** | **D** | **E** | **F** |
| **Number of defining Q-sorts** | | 13 | 17 | 11 | 11 | 12 | 12 |
| **Variance explained (%)** | | 9 | 13 | 8 | 9 | 9 | 9 |
| **Eigenvalue** | | 11.0 | 15.0 | 10.0 | 10.5 | 11.0 | 11.1 |
| **Items and scores** | | | | | | | |
| 1n | Effect of caring for patient on patients with same disease. | 5 | 3 | 4 | 6 | 4 | 3 |
| 2n | What continues the life patient has led. | 4 | 5 | 5 | 4 | 3 | 5 |
| 3 | Trying everything possible to save patient. | 9 | 8 | 6 | 8 | 9 | 8 |
| 4n | What promotes the life patient has valued.* | 5 | 5 | 6 | 5 | 6 | 5 |
| 5n | Effect of caring for patient on all patients in society. | 4 | 2 | 5 | 5 | 5 | 4 |
| 6n | The way patient used to make decisions. | 4 | 6 | 5 | 5 | 4 | 5 |
| 7n | Surrogate own religious or spiritual beliefs. | 2 | 4 | 7 | 3 | 2 | 7 |
| 8n | What surrogate would have wanted if in similar situation. | 5 | 4 | 4 | 3 | 6 | 3 |
| 9 | Family needs. | 6 | 7 | 6 | 6 | 7 | 5 |
| 10n | Surrogate own wishes for patient. | 6 | 4 | 5 | 2 | 5 | 4 |
| 11n | What patient requested in advance directives. | 6 | 6 | 6 | 6 | 4 | 6 |
| 12 | Patient pain and suffering. | 7 | 8 | 6 | 9 | 7 | 8 |
| 13n | Religious or spiritual beliefs of patient. | 3 | 7 | 8 | 5 | 3 | 7 |
| 14 | Surrogate burden due to patient care or disliked outcome. | 3 | 2 | 2 | 4 | 5 | 4 |
| 15n | What patient would have wanted based on past wishes. | 6 | 6 | 4 | 4 | 4 | 6 |
| 16 | Cost to society from caring for patient. | 3 | 1 | 4 | 6 | 3 | 2 |
| 17n | Financial concerns. | 2 | 5 | 3 | 4 | 8 | 2 |
| 18n | Life-long story of patient. | 4 | 4 | 3 | 2 | 2 | 3 |
| 19n | Reaching family agreement to distribute responsibility. | 5 | 5 | 5 | 7 | 5 | 5 |
| 20n | Reaching family agreement to maintain family cohesion. | 7 | 5 | 7 | 6 | 5 | 6 |
| 21 | Improving patient health.* | 8 | 9 | 8 | 8 | 8 | 9 |
| 22n | Medical facts. | 6 | 6 | 5 | 7 | 6 | 6 |
| 23 | Surrogate needs. | 1 | 3 | 1 | 1 | 1 | 1 |
| 24n | What patient wants now despite mental impairment. | 5 | 6 | 4 | 5 | 4 | 5 |
| 25n | Feeling of guilt because not trying everything possible. | 5 | 4 | 3 | 4 | 5 | 6 |
| 26 | Fear of loss of loved one. | 8 | 5 | 9 | 7 | 7 | 7 |
| 27n | Family burden due to patient care or disliked outcome. | 4 | 3 | 2 | 3 | 6 | 4 |
| 28n | What is in the best interests of patient. | 7 | 7 | 7 | 5 | 6 | 4 |

The six-factor solution accounted for 57% of the total variance and 76 (63%) of 120 Q-sorts (38 Q-sorts did not have significant loading and six were confounded). Item scores represent idealized scores of the 28 items for each program-generated model. Items numbers and abbreviated version are presented; full version is available in Additional file 1-Q-set items. “n” denotes neutral item on averaging-analysis (i.e., mean ranking score ˃4 and <6). “*” denotes consensus item on Q-methodology analysis. There were no differentiating items. Item scores correlation ranged from 0.30 (models C and E) to 0.71 (models B and F).
