# Supplementary material for: Factors underlying surrogate medical decision-making in middle eastern and east Asian women: a Q-methodology study
Source: BMC Palliat Care. 2020 Sep 1;19:137. doi: 10.1186/s12904-020-00643-9 (PMC7466416; doi:10.1186/s12904-020-00643-9)
Supplement: Supplementary file 5 — Additional file 5. Q-methodology technical report. [file 12904_2020_643_MOESM5_ESM.docx]

**Additional file 5-Q-methodology technical report**

We extracted 6 factors for ME respondents and 6 for EA respondents for each of the three perspectives. Extracting additional factors did not result in a substantial increase in the percentage of explained variance and further complicated factor interpretation. The factors were graphically rotated to reduce negative loading and confounding. Factor characteristics and item scores are presented in Tables 2S to 7S (Additional file 4-factor characteristics and item scores).

**Norm-perception (N-perspective) models in ME women**

Model A was unique in strongly disagreeing with the importance of “14.Surrogate burden due to taking care of patient or disliked outcome” and “25.Feeling of guilt because not trying everything possible.” It also strongly disagreed with “17.Financial concerns.” It can be classified as relatively surrogate’s burden-independent.

Model B was unique in strongly disagreeing with “10.Surrogate own wishes for patient.” It also strongly disagreed with “7.Surrogate own religious or spiritual beliefs” and “23.Surrogate needs” and disagreed with “14.Surrogate burden due to taking care of patient or disliked outcome.” It can be classified as relatively surrogate-independent (both burden/needs and preference).

Model C was unique in strongly disagreeing with “26.Fear of loss of loved one.” It also gave a rank of 4 to “25.Feeling of guilt because not trying everything possible.” On the other hand, it strongly disagreed with “18.Life-long story of patient,” disagreed with “6.The way patient used to make decisions,” and gave a rank of 4 to “2.What continues the life patient has led” and “4.What promotes the life patient has valued.” Further, it strongly disagreed with “9.Family needs,” disagreed with “20.Reaching family agreement to maintain family cohesion,” and assigned a rank of 4 to “Reaching family agreement to distribute responsibility.” It can be classified as relatively emotion, patient’s life-long narrative, and family needs-independent.

Model D was not unique in ranking any of the items. However, it strongly disagreed with “18.Life-long story of patient,” disagreed with “4.What promotes the life patient has valued,” and was neutral with “2.What continues the life patient has led” and “The way patient used to make decisions.” On the other hand, it strongly disagreed with “7.Surrogate own religious or spiritual beliefs” and disagreed with “8.What surrogate would have wanted if in similar situation.” It can be classified as relatively patient’s endorsed-life narrative and Golden rule-independent.

Model E was unique in strongly agreeing with “11.What patient requested in advance directives.” It gave a rank of 6 to “15.What patient would have wanted based on past wishes.” In addition, it strongly disagreed with “23.Surrogate needs” and “9.Family needs.” It can be classified as relatively patient’s preference-dependent.

Finally, model F was unique in strongly disagreeing with “1.Effect of caring for patient on patients with same disease.” It also strongly disagreed with “16.Cost to society from caring for patient” and “17.Financial concerns,” and gave a rank of 4 to “5.Effect of caring for the patient on all patients in society.” It can be classified as relatively society’s interests-independent.

The numbers of respondents who loaded only or with a higher loading score on models A, B, C, D, E, or F were 22, 14, 15, 10, 13, and 10, respectively (35 respondents did not have significant loading and one had equal significant loading on two factors).

**Patient’s perspective (P-perspective) models in ME women**

Model A was unique in strongly disagreeing with “26.Fear of loss of loved one” and gave a rank of 4 to “25.Feeling of guilt because not trying everything possible.” On the other hand, it strongly disagreed with “18.Life-long story of the patient,” disagreed with “2.What continues the life patient has led,” and gave a rank of 4 to “4.What promotes the life the patient has valued” and “6.The way patient used to make decisions.” It can be classified as relatively emotion and patient’s life-long narrative-independent.

Model B was not unique in ranking any of the items. However, it strongly disagreed with “17.Financial concerns,” “5.Effect of caring for patient on all patients in society,” and “16.Cost to society from caring for patient;” and disagreed with “1.Effect of caring for patient on patients with same disease.” In addition, it strongly agreed with “28.What is in the best interests of patient.” It can be classified as relatively cost and society’s interests-independent.

Model C was unique in strongly agreeing with “26.Fear of loss of loved one.” It also strongly agreed with “3.Trying everything possible to save patient.” On the other hand, it strongly disagreed with “5.Effect of caring for patient on all patients in society” and “16.Cost to society from caring for patient,” and disagreed with “1.Effect of caring for patient on patients with same disease.” It can be classified as relatively emotion-dependent and society’s interests-independent.

Model D was unique in strongly disagreeing with “13.Religious or spiritual beliefs of patient.” It also strongly disagreed with “7.Surrogate own religious or spiritual beliefs.” On the other hand, it was unique in strongly agreeing with “9.Family needs,” agreed with “20.Reaching family agreement to maintain family adhesion,” and gave a rank of 6 to “19.Reaching family agreement to distribute responsibility” and “27.Family burden due to taking care of patient or disliked outcome.” It can be classified as relatively religious/spiritual beliefs-independent and family needs-dependent.

Model E was not unique in ranking any of the items. However, it strongly disagreed with “25.Feeling of guilt because not trying everything possible” and disagreed with “26.Fear of loss of a loved one.” On the other hand, it agreed with “19.Reaching family agreement to distribute responsibility” and “20.Reaching family agreement to maintain family cohesion,” and gave a rank of 6 to “9.Family needs.” It can be classified as relatively emotion-independent and family needs-dependent.

Finally, model F was unique in strongly agreeing with “11.What patient requested in their advance directives” and “15.What patient would have wanted based on past wishes.” In addition, it strongly disagreed with “7.Surrogate’s own religious or spiritual beliefs”. It can be classified as relatively patient’s preference-dependent.

The numbers of respondents who loaded only or with a higher loading score on models A, B, C, D, E, or F were 14, 11, 16, 9, 12, and 18 (40 respondents did not have significant loading).

**Surrogate’s perspective (S-perspective) models in ME women**

Model A was unique in being neutral with “3.Trying everything possible to save patient” and in strongly agreeing with “11.What patient requested in advance directives” and “15.What patient would have wanted based on past wishes.” In addition, it strongly disagreed with “7.Surrogate own religious or spiritual beliefs” and “23.Surrogate needs.” It can be classified as relatively patient’s preference-dependent.

Model B was not unique in ranking any of the items. However, it strongly disagreed with “17.Financial concerns,” disagreed with “27.Family burden due to patient care or disliked outcome,” and strongly agreed with “26.Fear of loss of loved one.” It also strongly agreed with “3.Trying everything possible to save patient” and agreed with “25.Feeling of guilt because not trying everything possible.” On the other hand, it agreed with “7. Surrogate own religious or spiritual beliefs” and gave a rank of 6 to “13.Religious or spiritual beliefs of patient.” It can be classified as relatively emotion and religious/spiritual beliefs-dependent.

Model C was unique in strongly disagreeing with “5.Effect of caring for patient on all patients in society.” It also disagreed with “1.Effect of caring for patient on patients with same disease” and “16.Cost to society from caring for patient.” On the other hand, it strongly disagreed with “7.Surrogate own religious or spiritual beliefs” and “13.Religious or spiritual beliefs of patient.” It can be classified as relatively society’s interests and religious/spiritual beliefs-independent.

Model D was unique in strongly disagreeing with “6.The way patient used to make decisions.” In addition, it strongly disagreed with “18.Life-long story of patient” and assigned a rank of 4 to “2.What continues the life patient has led” and “4.What promotes the life patient has valued.” On the other hand, it was unique in strongly agreeing with “12.Patient pain and suffering” but gave only a rank of 6 to “28.What is in the best interests of patient.” It can be classified as relatively patient’s life-long narrative-independent.

Model E was unique in strongly agreeing with “28.What is in the best interests of patient.” It strongly agreed with “22.Medical facts” but gave only a rank of 6 to “12.Patient pain and suffering.” It was also unique in strongly disagreeing with “9.Family needs” and it strongly disagreed with “23.Surrogate needs.” On the other hand, it strongly disagreed with “13.Religious or spiritual beliefs of patient” and disagreed with “7.Surrogate own religious or spiritual beliefs.” It can be classified as relatively medical facts-dependent and religious/spiritual beliefs-independent.

Finally, model F was not unique in ranking any of the items. However, it strongly disagreed with “17.Financial concerns” and “16.Cost to society from caring for patient,” and disagreed with “1.Effect of caring for patient on patients with same disease” and “5.Effect of caring for patient on all patients in society.” It can be classified as society’s interests-independent.

The numbers of respondents who loaded only or with a higher loading score on models A, B, C, D, E, or F were 16, 24, 9, 10, 22, and 14 (23 respondents did not have significant loading and two had equal significant loading on two factors).

**Norm-perception (N-perspective) models in EA women**

Model A was unique in strongly disagreeing with “5.Effect of caring for patient on all patients in society.” It also strongly disagreed with “16.Cost to society from caring for patient” and disagreed with “1.Effect of caring for patient on patients with same disease.” Interestingly, although it was unique in strongly agreeing with “12.Patient pain and suffering,” it gave only a rank of 5 to “28.What is in the best interests of patient.” On the other hand, it strongly disagreed with “14.Surrogate burden due to taking care of patient or disliked outcome” and disagreed with “10.Surrogate own wishes for the patient” and “23.Surrogate needs.” It can be classified as relatively society’s interests and surrogate-independent.

Model B was unique in strongly disagreeing with “13.Religious or spiritual beliefs of patient.” It also strongly disagreed with “7.Surrogate own religious or spiritual beliefs.” On the other hand, it was unique in strongly disagreeing with “23.Surrogate needs” and it disagreed with “10.Surrogate own wishes for the patient” and “14.Surrogate burden due to taking care of patient or disliked outcome.” Further, it was unique in strongly agreeing with “26.Fear of loss of loved one” and it agreed with “3.Trying everything possible to save patient.” It can be classified as relatively religious/spiritual beliefs and surrogate-independent and emotion-dependent.

Model C was unique in strongly agreeing with “22.Medical facts.” It also agreed with “12.Patient pain and suffering” and “28.What is in the best interests of patient.” On the other hand, it disagreed with “2.What continues the life patient has led,” “11.What patient requested in advance directives,” and “13.Religious or spiritual beliefs of patient.” It can be classified as relatively medical facts-dependent and patient’s preference independent.

Model D was unique in strongly disagreeing with “17.Financial concerns” and “27.Family burden due to taking care of patient or disliked outcome.” On the other hand, it strongly disagreed with “16.Cost to society from caring for patient” and disagreed with “1.Effect of caring for patient on patients with same disease” and “5.Effect of caring for patient on all patients in society.” It can be classified as relatively financial needs and society’s interests-independent.

Model E was unique in strongly agreeing with “11.What patient requested in advance directives.” It also agreed with “24.What patient wants now despite mental impairment” and assigned ranks of 6 and 7 to “15.What patient would have wanted based on past wishes” and “13.Religiuous or spiritual beliefs of patient.” It also strongly disagreed with “10.Surrogate own wishes for patient.” On the other hand, it was unique in strongly disagreeing with “26.Fear of loss of loved one” and it disagreed with “25.Feeling of guilt because not trying everything possible.” It can be classified as relatively patient’s preference-dependent and emotion-independent.

Finally, model F was unique in strongly agreeing with “13.Religious and spiritual beliefs of patient.” However, it gave a rank of 4 to “15.What patient would have wanted based on past wishes,” a rank of 6 to “11.What patient requested in advance directives,” and a rank of 5 to “7.Surrogate own religious or spiritual beliefs.” It can be classified as relatively patient’s religious/spiritual beliefs-dependent.

The numbers of respondents who loaded only or with a higher loading score on models A, B, C, D, E, or F were 12, 10, 13, 18, 16, and 9, respectively (42 respondents did not have significant loading).

**Patient’s perspective (P-perspective) models in EA women**

Model A was unique in strongly disagreeing with “16.Cost to society from caring for patient” and “5.Effect of caring for patient on all patients on society.” It also strongly disagreed with “1.Effect of caring for patient on patients with same disease.” It can be classified as relatively society’s interests-independent.

Model B was unique in strongly disagreeing with “13.Religious or spiritual beliefs of patient” and “7.Surrogate own religious or spiritual beliefs.” It can be classified as relatively religious/spiritual beliefs-independent.

Model C was unique in strongly agreeing with “17.Financial concerns.” It also agreed with “9.Family needs.” On the other hand, it strongly disagreed with “26.Fear of loss of loved one” and disagreed with “25.Feeling of guilt because not trying everything possible.” It can be classified as relatively financial needs-dependent and emotion-independent.

Model D was unique in strongly disagreeing with “25.Feeling of guilt because not trying everything possible.” It also strongly disagreed with “26.Fear of loss of loved one.” On the other hand, it was unique in strongly agreeing with “9.Family needs,” it agreed with “17.Financial concerns,” and it assigned a rank of 6 to “19.Reaching family agreement to distribute responsibility,” “20.Reaching family agreement to maintain family cohesion,” and “27.Family burden due to taking care of patient or disliked outcome.” It can be classified as relatively emotion-independent and family’s needs-dependent.

Model E was unique in strongly disagreeing with “15.What patient would have wanted based on past wishes.” It also disagreed with “4.What promotes the life patient has valued” and gave a rank of 4 to “11.What patient requested in advance directives” and “2.What continues the life patient has led.” On the other hand, it strongly agreed with “13.Religious or spiritual beliefs of patient” but gave a rank of 5 to “7.Surrogate own religious or spiritual needs.” It can be classified as relatively patient’s preference-independent and patient’s religious/spiritual beliefs-dependent.

Finally, model F was unique in strongly disagreeing with “6.The way patient used to make decisions” and “11.What patient requested in advance directives.” It also disagreed with “18.Life-long story of patient” and gave a rank of 5 to “15.What patient would have wanted based on past wishes.” It can be classified as relatively patient’s preference-independent.

The numbers of respondents who loaded only or with a higher loading score on models A, B, C, D, E, or F were 18, 12, 11, 8, 12, and 8 (51 respondents did not have significant loading).

**Surrogate’s perspective (S-perspective) models in EA women**

Model A was not unique in ranking any of the items. However, it strongly disagreed with “7.Surrogate own religious or spiritual beliefs” and disagreed with “13.Religious or spiritual beliefs of patient.” On the other hand, it strongly disagreed with “17.Financial concerns” and disagreed with “16.Cost to society from caring for patient.” It can be classified as relatively religious/spiritual beliefs and financial needs-independent.

Model B was unique in strongly disagreeing with “5.Effect of caring for patient on all patients in society.” In addition, it strongly disagreed with “16.Cost to society from caring for patient” and disagreed with “1.Effect for caring for patient on patients with same disease.” It can be classified as relatively society’s interest-independent.

Model C was unique in strongly disagreeing with “27.Family burden due to taking care of patient or disliked outcome.” In addition, it strongly disagreed with “14.Surrogate burden due to taking care of patient or disliked outcome” and disagreed with “17.Financial concerns.” On the other hand, it was unique in strongly agreeing with “13.Religious or spiritual beliefs of patient” and it agreed with “7.Surrogte own religious or spiritual beliefs.” It can be classified as relatively family burden-independent and religious/spiritual beliefs-dependent.

Model D was unique in strongly disagreeing with “10.Surrogate own wishes for patient.” It also disagreed with “8.What surrogate would have wanted if in similar situation” and “7.Surrogate own religious or spiritual beliefs.” It can be classified as relatively surrogate’s preference independent.

Model E was unique in strongly agreeing with “17.Financial concerns.” It also agreed with “9.Family needs.” On the other hand, it strongly disagreed with “18.Life-long story of patient,” disagreed with “2.What continues the life patient has led” and “13.Religious or spiritual beliefs of patient,” and assigned a rank of 4 to “6.The way patient used to make decisions.” It can be classified as relatively financial needs-dependent and patient’s authentic-life narrative-independent.

Finally, model F was not unique in ranking any of the items. However, it strongly disagreed with “16.Cost to society from caring for patient” and “17.Financial concerns.” It also disagreed with “1.Effect of caring for patient on patients with same disease” and assigned a rank of 4 to “5.Effect of caring for patient on all patients in society.” On the other hand, it agreed with “7.Surrogate own religious or spiritual beliefs” and “13.Religious or spiritual beliefs of patient.” It can be classified as relatively society’s interests-independent and religious/spiritual beliefs-dependent.

The numbers of respondents who loaded only or with a higher loading score on models A, B, C, D, E, or F were 13, 19, 12, 12, 13, 12 (38 respondents did not have significant loading and one had equal significant loading on two factors).
